# TSC2 S1365A mutation potently regulates CD8^+^ T cell function and differentiation and improves adoptive cellular cancer therapy

**DOI:** 10.1172/jci.insight.167829

**Published:** 2023-11-08

**Authors:** Chirag H. Patel, Yi Dong, Navid Koleini, Xiaoxu Wang, Brittany L. Dunkerly-Eyring, Jiayu Wen, Mark J. Ranek, Laura M. Bartle, Daniel B. Henderson, Jason Sagert, David A. Kass, Jonathan D. Powell

**Affiliations:** 1 Bloomberg-Kimmel Institute for Immunotherapy,; 2 Division of Cardiology, Department of Medicine, and; 3 Department of Pharmacology and Molecular Sciences, Johns Hopkins University School of Medicine, Baltimore, Maryland, USA.; 4 CRISPR Therapeutics, South Boston, Massachusetts, USA.

**Keywords:** Cell Biology, Immunology, Adaptive immunity, Cancer immunotherapy, T cells

## Abstract

MTORC1 integrates signaling from the immune microenvironment to regulate T cell activation, differentiation, and function. TSC2 in the tuberous sclerosis complex tightly regulates mTORC1 activation. CD8^+^ T cells lacking TSC2 have constitutively enhanced mTORC1 activity and generate robust effector T cells; however, sustained mTORC1 activation prevents generation of long-lived memory CD8^+^ T cells. Here we show that manipulating TSC2 at Ser1365 potently regulated activated but not basal mTORC1 signaling in CD8^+^ T cells. Unlike nonstimulated TSC2-KO cells, CD8^+^ T cells expressing a phosphosilencing mutant TSC2-S1365A (TSC2-SA) retained normal basal mTORC1 activity. PKC and T cell receptor (TCR) stimulation induced TSC2 S1365 phosphorylation, and preventing this with the SA mutation markedly increased mTORC1 activation and T cell effector function. Consequently, SA CD8^+^ T cells displayed greater effector responses while retaining their capacity to become long-lived memory T cells. SA CD8^+^ T cells also displayed enhanced effector function under hypoxic and acidic conditions. In murine and human solid-tumor models, SA CD8^+^ T cells used as adoptive cell therapy displayed greater antitumor immunity than WT CD8^+^ T cells. These findings reveal an upstream mechanism to regulate mTORC1 activity in T cells. The TSC2-SA mutation enhanced both T cell effector function and long-term persistence/memory formation, supporting an approach to engineer better CAR-T cells for treating cancer.

## Graphical abstract

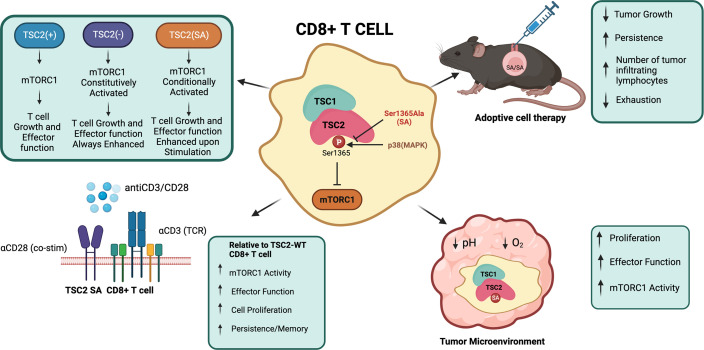

## Introduction

The mechanistic target of rapamycin (mTOR) is an evolutionarily conserved serine/threonine kinase that integrates cues from the environment to regulate cellular growth (1). In T-lymphocytes and throughout the immune system, mTOR is a potent regulator of cell activation, differentiation, and function (2, 3). This is achieved by mTOR’s strong ability to control metabolic programming, cell growth, proliferation, and survival (4). MTOR signals via 2 multiprotein complexes, mTORC1 and mTORC2, that differentially regulate T cells (5). For CD4^+^ T cells, signaling downstream of mTORC1 promotes Th1 and Th17 differentiation, while mTORC2 regulates Th2 differentiation (6, 7). Interestingly, antigen recognition in the absence of mTOR signaling leads to the generation of Tregs (8, 9). For CD8^+^ T cells, mTORC1 activation is critical for effector cell generation and function (10, 11), while its inhibition promotes the generation of memory CD8^+^ T cells (12). mTORC2 ablation in CD8^+^ T cells also promotes memory T cell generation but does not inhibit effector T cells (10).

mTORC1 activity is tightly regulated upstream by the tuberous sclerosis complex (TSC), which consists of hamartin (TSC1) and tuberin (TSC2) (13, 14). TSC2 is a GTPase activating protein (GAP) for the small GTPase Ras homolog enriched in brain (Rheb) residing on the lysosomal surface along with the mTORC1 complex, and TSC is a constitutive inhibitor of mTORC1 in a Rheb-dependent manner. TSC2 activity is potently influenced by phosphorylation of multiple residues on TSC2 that are mediated by distinct kinases to alter mTORC1 activity based on various environmental signals. Most prominently, the stimulation of the PI3K/PDK1/AKT/TSC2 axis by growth factors or T cell activation activates mTORC1 signaling through inhibitory phosphorylation of TSC2 at serine 939 to allow Rheb to activate mTORC1 through still-unknown mechanisms (13). An equally potent TSC inhibitory effect is conferred by ERK1/2 phosphorylation at S540/S664 (15). Alternatively, increased AMP levels indicative of metabolic stress activates AMP activated protein kinase (AMPK) phosphorylating TSC2 at T1271/S1387, thus enhancing its ability to suppress mTORC1 signaling (16). Also, under environmental hypoxic stress, Regulated in development and DNA damage response-1 (REDD1) induction competitively binds to 14-3-3 proteins, releasing the cytosolic sequestration of the TSC1/2 complex to inhibit mTORC1 activity (17).

Consistent with the role of mTORC1 in promoting effector CD8^+^ T cells, TSC2 KO (TSC2^–/–^) CD8^+^ T cells with constitutive elevated mTORC1 activity demonstrate enhanced effector function and display superior antitumor activity (10). However, TSC2^–/–^ CD8^+^ T cells fail to convert to long-lived memory T cells due to their inability to tone down mTORC1 signaling, thus driving cells to terminal differentiation. Recently, phosphorylation of TSC2 at Serine 1365 (S1365 in mice; S1364 in human) was shown to be a critical site for the negative regulation of mTORC1 in stressed cardiac myocytes, fibroblasts, and intact hearts (18–20). Mutation of this site from serine to an alanine (SA) to prevent its phosphorylation resulted in increased mTORC1 activation but only during states of pathological stress. Importantly, unlike TSC2^–/–^ cells, which typically demonstrate constitutive mTORC1 activity, TSC2 SA cells and mice with a SA–knock-in phosphosilencing mutation did not display increased basal mTORC1 activity. Similar phenotypes were observed in heterozygous and homozygous SA-KI mice, indicating an autosomal dominant effect by possessing just 1 mutant copy. Mutating the same serine to glutamic acid to create a phosphomimetic (S1365E or SE) also unaltered basal mTORC1 activity but attenuated mTORC1 activation upon stress. MTORC1-stimulation pathways particularly regulated by the phospho-state of S1365 (or its genetic substitution with alanine or glutamic acid) are mediated by mitogen activated kinases, including ERK1/2, whereas Akt activation of mTORC1 appears unaltered by the status of S1365 (19). While several kinases can result in TSC2 1365 phosphorylation, including protein kinase C and cGMP-stimulated kinase-1 (cGK-1), we identified the latter relevant for suppressing mTORC1 coactivation in cardiomyocytes. The role of this signaling node in T cells or other immune cells remains unknown.

Given these prior observations, we speculated that phosphorylation of S1365 on TSC2 may also potently regulate activation-induced mTORC1 activity in T cells. Furthermore, we hypothesized that the SA silencing mutation at this site might promote enhanced effector CD8^+^ T cell generation due to increased mTORC1 activity immediately upon activation while retaining their ability to become long-lived memory T cells, with mTORC1 activity eventually returning to basal levels. Here, we reveal a role for S1365 in regulating mTORC1 activity in T cells to generate T cells with enhanced replicative capacity, effector function, persistence, and antitumor activity.

## Results

### TSC2 constitutively suppresses mTORC1 activity and inhibits T cell effector cell generation.

**Figure 1:**
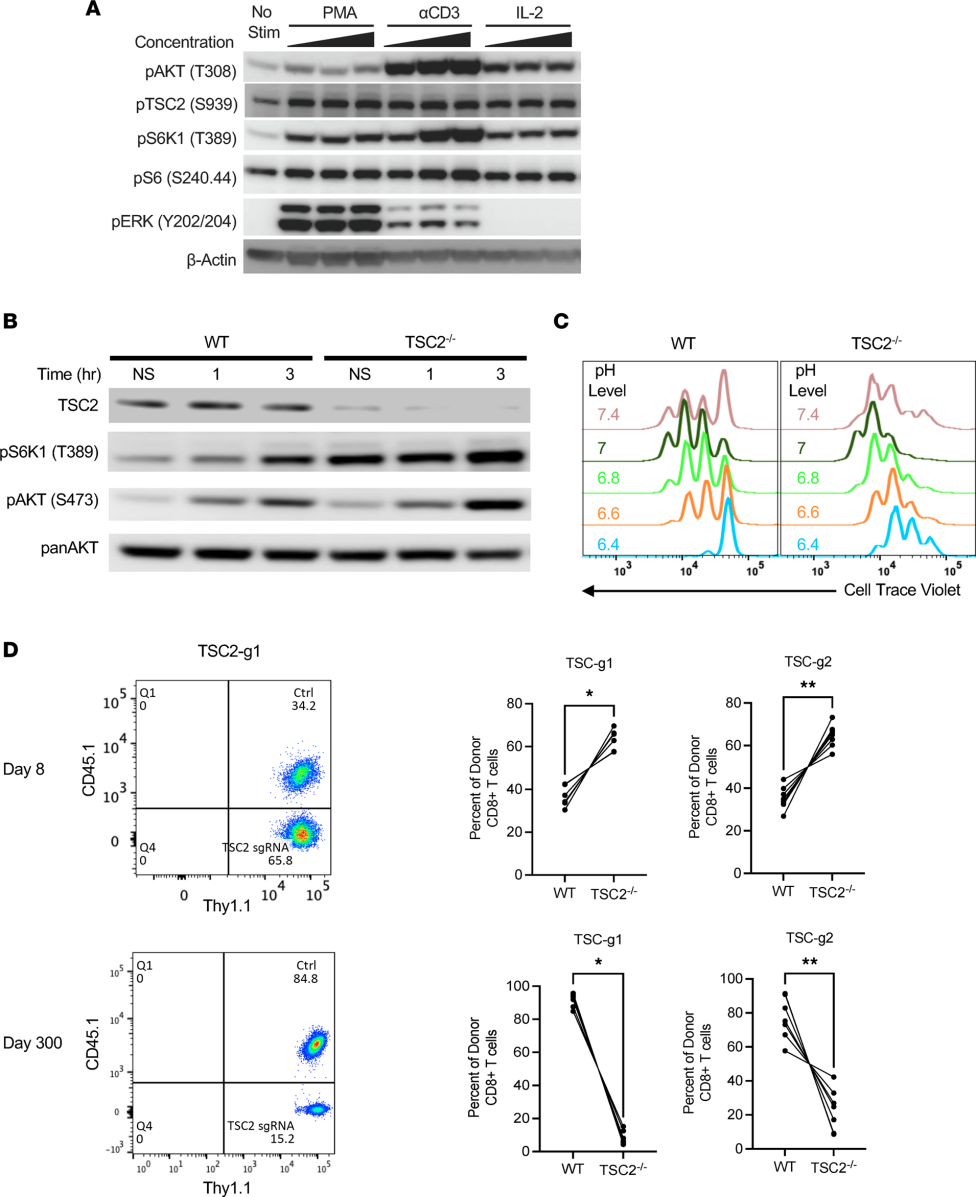
TSC2 is a central environmental hub that modulates mTORC1 activity and differentiation in T cells. (**A**) Immunoblot analysis of the PI3K/AKT/TSC2/mTORC1 pathway in resting T cells upon dose dependent stimulation of different stimuli for 30 minutes. PMA: phorbol ester, αCD3, IL-2. (**B**) WT and TSC2^–/–^ CD8^+^ T cells were stimulated via TCR and Co-Stim with agonists anti-CD3 and anti-CD28 for 1 and 3 hours, and mTORC1 and mTORC2 activity was measured by immunoblot. The 0 hour indicates baseline with no simulation. (**C**) Flow cytometry proliferation analysis of WT and TSC2^–/–^ CD8^+^ T cells stimulated and grown in different pH level media conditions. Data are measured on day 3. (**D**) Different congenic naive WT P14 (gp33^+^) CD8^+^ T cells were isolated and CRISPR edited using with CAS9 and a control sgRNA (Ctrl) or 1 of 2 sgRNAs (g1, or g2) targeting TSC2. Ctrl and TSC2 sgRNA–edited CD8^+^ T cells were mixed at 1:1 ratio and coadoptively transferred into naive WT recipients and infected with LCMV Armstrong for acute (day 8, blood) and memory analysis of donor CD8^+^ T cells (spleen) via flow cytometry. *n* = 5/group for guide 1 (g1); *n* = 6/group for guide 2 (g2); *P < 0.05, **P < 0.01 paired t test. Data are representative of 3 or more independent experiments (**A**–**C**), and 2 independent exp (**D**).

In the canonical mTORC1 signaling pathway, environmental cues lead to PI3K and PDK1 activation, which leads to the phosphorylation (T308) and activation of AKT, which in turn leads to the phosphorylation and inactivation of TSC2 (S939). CD8^+^ T cells activated by T cell receptor (TCR) engagement by anti-CD3 (αCD3); PMA to activate PKC, which is downstream of the TCR pathway; or IL-2 displayed acute Akt and TSC2 phosphorylation and corresponding activation of mTORC1 reflected by increased phosphorylation of p70S6K (S6K1) and ribosomal S6 (Figure 1A). PMA and TCR, but not IL-2, also led to ERK1/2 phosphorylation. TSC2^-/-^ CD8^+^ T cells had increased constitutive mTORC1 activity and also amplified TCR-induced activity (Figure 1B) as previously reported (10). Moreover, TSC2^–/–^ CD8^+^ T cells proliferated more readily in normal but also more stressful (e.g., acidic) conditions, emphasizing TSC2’s ability to sense environmental cues to modulate mTORC1 activity (Figure 1C). As previously shown, TSC2^–/–^ CD8^+^ T cells respond to an acute infection with greater proliferation in vivo as compared with WT T cells. To confirm our earlier findings, we used TSC2^–/–^ P14 CD8^+^ T cells that recognize lymphocytic choriomeningitis virus (LCMV) antigen (generated by CRISPR editing) in naive T cells (2 different CRISPR-Cas9 guides g1 and g2 employed). An equal number of control or TSC2^–/–^ edited P14 CD8^+^ T cells (e.g., 1:1 ratio) were transferred into naive WT recipient mice, and their response was tracked following acute LCMV Armstrong infection. The percent of the respective and equal donor cells CD8^+^ T cells were identified based on different surface congenic markers (Supplemental Figure 1; supplemental material available online with this article; https://doi.org/10.1172/jci.insight.167829DS1). On day 8 of the peak acute response to LCMV, TSC2^–/–^ CD8^+^ T cells had proliferated more robustly versus control T cells (Figure 1D). However, at day 300 after infection, there were far fewer memory TSC2^–/–^ CD8^+^ T versus controls. Thus, while the CD8^+^ T cell effector response (mTORC1 activity and proliferation) was enhanced by genetic deletion of TSC2, chronic mTORC1 signaling prevented the generation of long-term memory T cells. This highlights the key therapeutic limitation of genetically deleting TSC2 in CD8^+^ T cell, since sustained mTORC1 compromises memory cell formation critical for proper long-term immunity.

### TSC2 S1365 is phosphorylated in T cells.

**Figure 2:**
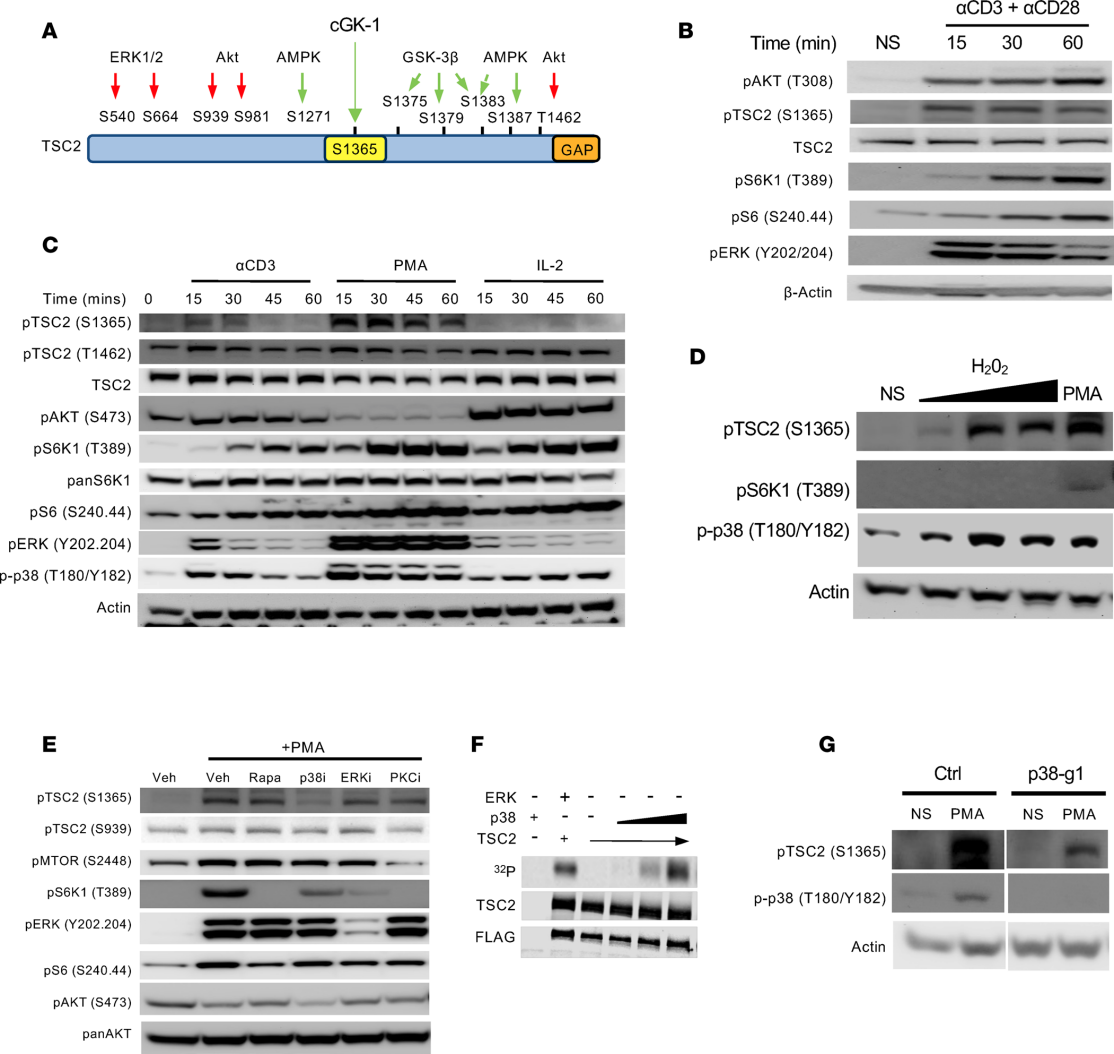
Identification of a TSC regulatory node (S1365) in T cells. (**A**) TSC2 protein map shows S1365 relative to other known activating (green) and inhibitory (red) phosphorylation sites for the mouse protein. The human protein is highly homologous in this region, but S1365 in mice is S1364 in human. (**B**) Immunoblot analysis of T cells before and after stimulation with TCR agonist (αCD3) + Co-Stim with αCD28 after 15, 30, or 60 minutes. (**C**) Similar study as in **B** but using αCD3 stimulus alone versus PMA or IL-2. (**D**) T cells were exposed to increasing concentrations (0.01 mM, 0.1 mM, 1 mM) of H_2_O_2_ for 30 minutes and assayed by immunoblot analysis as indicated. PMA is a positive control. (**E**) T cells were stimulated with PMA in the presence of vehicle (Veh), rapamycin, a selective p38 inhibitor (p38i), ERK inhibitor (ERKi), or PKC inhibitor (PKCi). The p38 inhibitor and, to a lesser extent, the PKC inhibitor blocked phosphorylation at TSC2 (S1365). (**F**) Pulldown experiment using Flag tagged TSC2 protein expressed in TSC2 KO HEK 293T cells and IP: Flag. Immune complexes incubated with ERK (positive control) or increasing concentration of active p38 and ^32^P-ATP. The ^32^P autoradiography band shows label incorporation in TSC2 by both ERK and p38. Total TSC2 detected by Ab-TSC2 or FLAG shown below. (**G**) Effect of gene silencing (g1) of p38α by CRISPR-CAS9 in naive T cells subsequently activated with combined αCD3 and αCD28 and expanded with IL-2. Immunoblots are from CD8^+^ T cells isolated on day 7 and stimulated with PMA for 30 minutes. White line denotes separation of lanes from the same gel comparing control (Ctrl) and p38 KO (g1) conditions. Data are representative of at least 3 independent experiments (**B**–**E**), and 2 independent experiments (**G**).

S1365 resides in the region of TSC2 that contains multiple phosphorylation sites modified by various kinases that result in greater TSC suppression of mTORC1 (green arrows, Figure 2A) (18). Inhibiting phosphorylation at this site by mutating serine to alanine (TSC2 SA or SA) led to a marked increase in stimulated mTORC1 activity in cardiac myocytes (18). However, unlike TSC2^–/–^, which results in constitutive mTORC1 activation (Figure 1B), expression of the SA-mutant did not alter basal mTORC1 signaling but only increased upon stimulation. Furthermore, in cardiac myocytes, it was found that cGK-1 was responsible for selective phosphorylation of TSC2 at this serine residue (18).

Based on these prior findings, we speculated that TSC2 S1365 plays a similar biological role to regulate mTORC1 activity in T cells. To test this, we stimulated CD8^+^ T cells with αCD3 + αCD28 and found phosphorylation of TSC2 at S1365 in as early as 15 minutes after T cell stimulation (Figure 2B). At this time, S6K1 and S6 were minimally activated despite activation of both Akt and ERK1/2, but mTORC1 activation was observed shortly afterward. These phosphorylation kinetics are consistent with TCR-coupled mTORC1 signaling that simultaneously promotes phosphorylation of TSC2 S1365. This is analogous to increased pS1365 reported in cardiac myocytes and fibroblasts exposed to pathological growth factor stimuli or cardiac hemodynamic stress in vivo (18). As shown in Supplemental Figure 2, A and B, pS1365 was not observed in T cells expressing either the SA/SA, an alternative SE/SE mutation, or TSC2 deletion, confirming specificity of the immunoblot antibody and signal despite other nearby residues known to be phosphorylated on TSC2 (Figure 2A).

We next tested if other mTORC1 activators also phosphorylate pS1365 in T cells. We found rapid robust phosphorylation in response to PMA, which activates PKC and, in turn, downstream kinases such as ERK1/2 and p38 — all pathways actively engaged upon TCR stimulation (21) (Figure 2C). However, we did not observe this result with IL-2 stimulation — a cytokine that canonically signals through the PI3K/AKT pathway — even at high concentrations (Supplemental Figure 2C). Thus, while all 3 stimuli activate mTORC1 (TCR and IL-2 also activating mTORC2 reflected by Akt S473 phosphorylation), IL-2 did not result in TSC2 S1365 phosphorylation. IL-2 led to more Akt phosphorylation of TSC2 (T1642) without activating ERK1/2. These findings are consistent with a recent study in other cell types where we found the latter but not former results in phosphorylation at pS1365 and, in turn, mTORC-1 regulation (19). S1364 (the analogous residue in human TSC2) phosphorylation was also observed with both TCR-induced activation and PMA in human CD8^+^ T cells (Supplemental Figure 2D). We also found that exposure to H_2_O_2_ increases pS1365 in parallel with p38 activation (a known H_2_O_2_ target), although H_2_O_2_ alone had no effect on mTORC1 activity (Figure 2D). A similar rise in p38 and TSC2 S1365 phosphorylation with H_2_O_2_ exposure was observed in human T cells (Supplemental Figure 2E). Together, these results establish for the first time to our knowledge that, in T cells, TSC2 S1365 is phosphorylated by both select extrinsic immune-activating signals (TCR engagement and PMA [PKC], but not IL-2) as well as extrinsic environmental cues such as H_2_O_2_.

We first showed that, in cardiomyocytes, TSC2 S1365 is phosphorylated by cGK-1 (18). However, cGK-1 agonism by cGMP or inhibition with DT3 had no effect on pS1365 in T cells (Supplemental Figure 3A). Moreover, public databases for RNA expression (Immgen; ref. 22) and proteomics (ImmPRes) (http://immpres.co.uk/) reveal that cGK-1 mRNA and protein level are not basally expressed in CD8^+^ T cells nor in other immune cells other than mast cells (Supplemental Figure 3, B and C). To identify alternative kinases responsible for S1365 phosphorylation in T cells, cells were stimulated with PMA and coincubated with rapamycin (testing if downstream mTORC1 factors contributed) or selective p38, ERK1/2, or PKC inhibitors. mTORC1 activity (pS6K1) declined with all inhibitors, yet only p38 blockade lowered pS1365 (Figure 2E). Using a radiolabeled ATP kinase assay with recombinant p38 and TSC2, we found a dose-dependent increase in TSC2 phosphorylation by p38, demonstrating p38’s direct ability to regulate TSC2 (Figure 2F). Lastly, we used CRISPR to delete p38a in naive T cells (guide, g1). This markedly reduced pS1365 in response to PMA (Figure 2G). Together, these data show that TSC2 S1365 was phosphorylated in T cells in response to various T cell activation mechanisms that couple to MAP kinase stimulation in a p38-dependent manner.

### TSC2 S1365A phosphomutant regulates T cell function.

To test the functional consequences of expressing the SA phospho-silencing mutation in T cells, we studied primary CD8^+^ T cells obtained from global SA knockin (KI) mice (18). Importantly, there were no significant differences in thymic T cell populations comparing TSC2 WT and TSC2 SA/SA mice (Supplemental Figure 4, A and B). We also found peripheral CD4^+^ and CD8^+^ T cell percentages in the spleen to be similar among these groups, whereas percentages of CD8^+^ T cells declined and CD4^+^ T cells increased in TSC2^–/–^ mice (Supplemental Figure 4C). Importantly, consistent with our previously reported (10) sustained mTORC1 activity even at rest in TSC2^–/–^ CD8^+^ T cells (e.g. Figure 1B), these cells but not SA cells also exhibited an elevated activation profile (CD62L^lo^CD44^hi^) under basal conditions (Supplemental Figure 4D).

**Figure 3:**
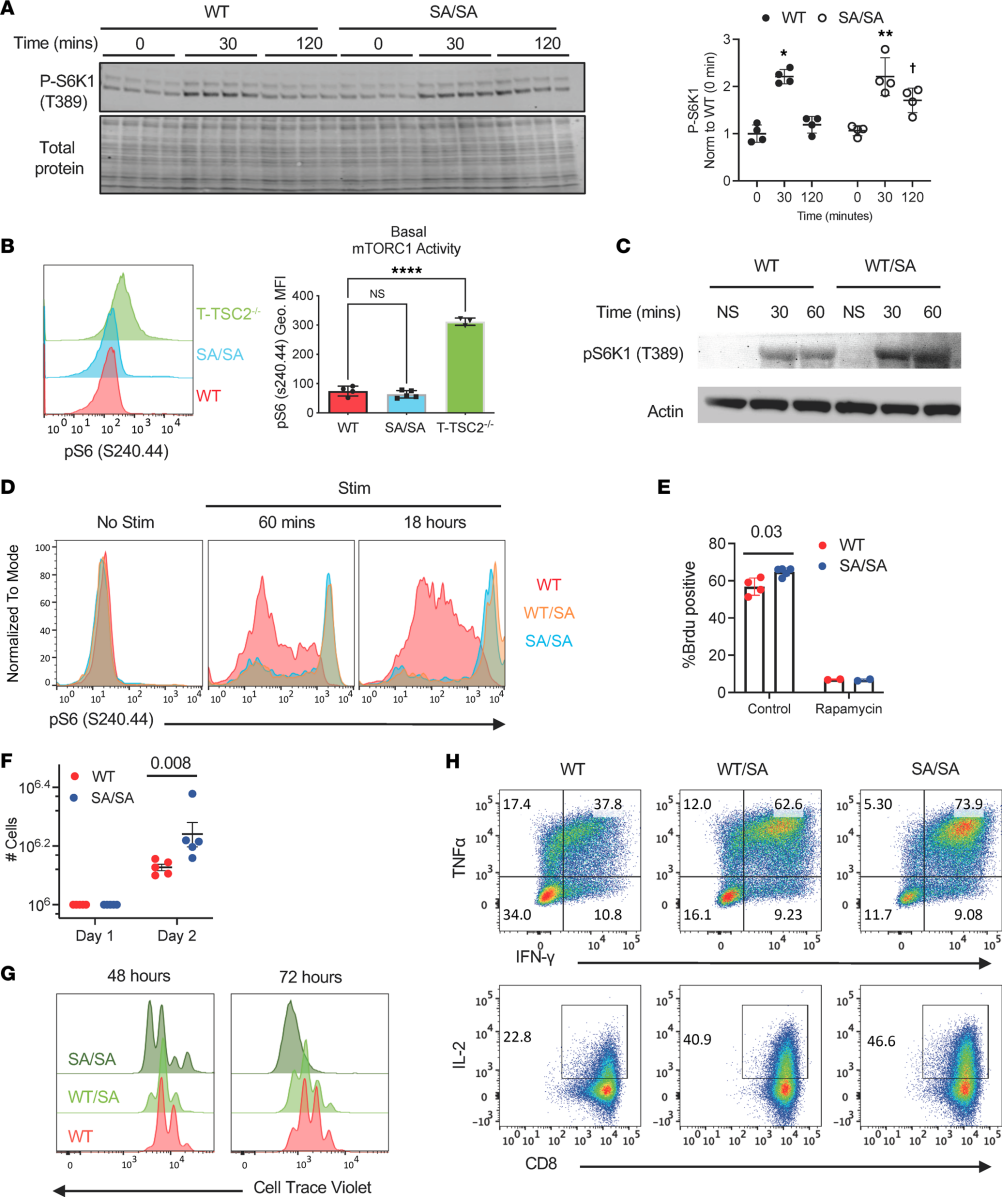
Mutating TSC2 at S1365 augments mTORC1 activity and function in murine CD8^+^ T cells upon stimulation. (**A**) Immunoblot analysis of mTORC1 activity at baseline (time 0) and 0.5 and 2 hours after stimulation with αCD3/αCD28 in CD8^+^ T cells expressing WT or TSC2 SA/SA. Summary data to the right (*n* = 4/group); 2-way ANOVA, Sidaks multiple comparisons test: * *P* < 0.00003; ** P ≤ 0.003 versus Time 0, 120 min; †*P* = 0.012 versus WT at 120 min). (**B**) mTORC1 activity assessed by intracellular staining for mTORC1 activity via phospho-S6 levels in naive WT, TSC2 WT, SA/SA, or TSC2^–/–^ CD8^+^ T cells. Geo-MFI, geometric mean fluorescence intensity. Summary data to right; *n* = 4, 5, 3 for groups left to right; 1-way ANOVA, ****P = 3 × 10^–11^, Holm-Šídáks multiple-comparisons test. (**C**) Example immunoblot for mTORC1 activation (pS6K-1) from similar experiment as in **A** but with TSC2 WT/SA CD8^+^ T cells. (**D**) Naive WT, WT/SA, and SA/SA CD8^+^ T cells stimulated with αCD3 and αCD28 to activate TCR signaling for 60 minutes and 18 hours and mTORC1 activity assessed by intracellular staining for mTORC1 activity via phospho-S6 levels. (**E**) WT and SA/SA CD8^+^ T cells stimulated with αCD3 and αCD28 with or without rapamycin to inhibit mTOR, and cell cycle entry assessed by BrdU^+^ staining by flow cytometry. *n* = 4 biologic replicates, 5 control, 2 rapamycin; *P* value Kruskal Wallis test. (**F**) Same experiment (without rapamycin) with cell counts measured after 24 and 48 hours. *n* = 5/group; significance found with Kruskal Wallis test. (**G**) WT, WT/SA, and SA/SA mutant OTI CD8^+^ T cells stimulated with OVA I peptide and cell proliferation analyzed by flow cytometry on day 2 and day 3 after activation. (**H**) CD8^+^ T cells from WT, WT/SA, and SA/SA genotypes, activated and expanded in IL-2, and then examined for cytokine analysis of IFN-γ, TNF-α, and IL-2 after restimulating with αCD3/αCD28. Data are representative of at least 2 independent experiments.

We next isolated WT and SA/SA CD8^+^ T cells and stimulated them with αCD3 (TCR) or αCD3 + αCD28 (Co-Stim), measuring pS6K1. Resting CD8^+^ T cells expressing SA/SA displayed no constitutive S6K1 phosphorylation, but this increased after TCR/Co-Stim and was more sustained at a higher level compared with WT T cells (Figure 3A). Basal mTORC1 activity was also measured by flow cytometry using pS6 (S240) as a readout; this was unaltered in SA/SA CD8^+^ T cells in contrast to an order of magnitude elevation in TSC2^–/–^ T cells (Figure 3B). Our initial studies identified the SA mutation acting as an autosomal dominant (18) in cardiomyocytes, so we next tested the effect of a WT/SA mutation. The heterozygote mutation also induced marked augmentation of mTORC1 activity upon TCR/Co-Stim (Figure 3C). This was further confirmed by flow cytometry showing that resting WT, WT/SA, and SA/SA CD8^+^ T cells have minimal mTORC1 activity, but after TCR/Co-stim, both WT/SA and SA/SA T cells exhibited a 2–order of magnitude rise in pS6 phosphorylation (Figure 3D). To test if SA/SA CD8^+^ T cells displayed faster cell cycle entry over WT CD8^+^ T cells, BrdU incorporation was measured in T cells 24 hours after TCR activation; it was higher in SA/SA T cells (Figure 3E). As expected, rapamycin near fully reduced cell cycle entry in both groups. In addition, T cell proliferation was greater after 24 hours (by cell counting), 48 hours, and 72 hours (by cell violet dilution assay) in cells expressing SA/SA (Figure 3, F and G), with a dose-response effect between WT/SA and SA/SA measured at 48 and 72 hours (Figure 3G). CD8^+^ T cell effector function was measured using IFN-γ, TNF-α, and IL-2 expression upon restimulation. Concordant with greater proliferation and mTORC1 activity, WT/SA and SA/SA CD8^+^ T displayed more effector function over WT T cells, the highest being in SA/SA T cells (Figure 3H). These data demonstrate the biologic effect of preventing TSC2 S1365 phosphorylation by an SA mutation on TCR-induced mTORC1 activity and, thus, proliferation and effector function.

### TSC2 SA T cells demonstrate enhanced effector and memory generation in vivo.

**Figure 4:**
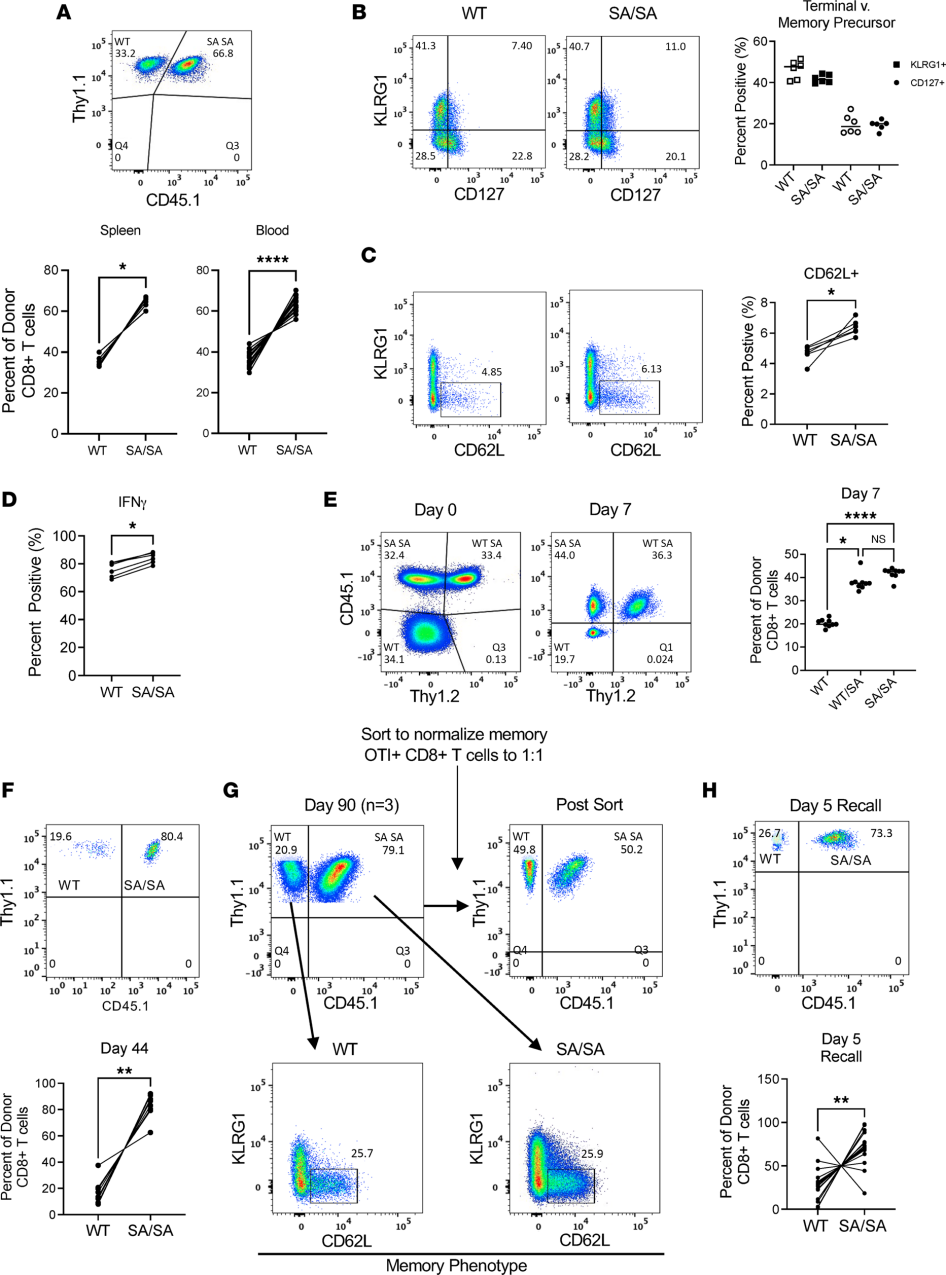
CD8^+^ T cells expressing TSC2 SA mutation have greater effector function while still preserving memory formation and recall capacity. WT and mutant TSC2 SA/SA transgenic CD8^+^ T cells were coadoptively transferred (1:1) into naive WT hosts followed with acute pathogen infection. (**A**) Flow cytometry plot of transferred OTI CD8^+^ T cells (top) and summary data (bottom) showing percent of WT versus SA/SA genotype from donor population 8 days after exposure to LM-OVA in spleen (*n* = 6) and blood (*n* = 17). *P < 0.05, ****P < 0.0001 paired 2-tailed *t* test. (**B**–**D**) Phenotypic and functional analysis of donor WT and mutant TSC2 SA/SA CD8^+^ T cells during acute infection from **A**. * *P* < 0.05, *n* = 6. (**E**) Triple coadoptive transfer of WT, WT/SA, or SA/SA TSC2 OTI CD8^+^ T cells into mice then infected with LM-OVA with relative number of cells in each group identified 1 week later. *n* = 9/group; *P < 0.05, ****P < 0.0001, 1-way ANOVA, Sidak’s multiple-comparison test. (**F**) Percent present of donor WT versus mutant TSC2 SA/SA CD8^+^ T cells 44 days after injection (blood, memory phase). *n* = 9; **P = 0.004, Wilcoxon 2-tailed signed-rank test. (**G**) Naive mice received equal cotransfer of memory OTI CD8^+^ WT or SA expressing T cells for recall with LM-OVA. At day 90, the percent of memory WT and mutant TSC2SA OTI CD8^+^ T cells in spleen (top, combined *n* = 3) and characterized based on KLRG1 (–) and CD62L (+) for memory phenotype (lower panels). These sorted memory CD8^+^ T cells were then injected in equal numbers (1:1) to naive WT recipients (top, right) who were subsequently infected with LM-OVA to assess memory recall ability. (**H**) After 5-days after infection, graphical summary of donor WT and SA/SA CD8^+^ T cells in spleen. **P = 0.003 Wilcoxon, *n* = 16. Data are representative of at least 3 independent experiments except **E**, with 2.

Based on these in vitro observations, we next tested the effector response of CD8^+^ T cells expressing TSC2 SA CD8^+^ T cells in vivo. Differentially identifiable congenic donor WT or SA/SA-OTI (TCR recognizing OVA) CD8^+^ T cells were coadoptively transferred 1:1 into naive WT recipients that were then infected with listeria-OVA (LM-OVA) to induce an acute immune response (Supplemental Figure 5A). On day 8, we analyzed the relative percent of WT versus SA/SA expressing CD8^+^ donor T cells in the spleen and blood. As observed in vitro, there was a greater percentage of SA/SA versus WT CD8^+^ T cells, demonstrating their enhanced proliferation in response to an acute infection (Figure 4A). Interestingly, there were no noticeable phenotypic differences (terminal [KLRG1^+^] versus memory precursors [CD127^+^]) between WT and SA/SA CD8^+^ T cells; however, there was a slight increase in central memory T cells as defined by CD62L^+^KLRG1^–^ expression (Figure 4, B and C). We also found enhanced effector function in SA/SA CD8^+^ T cells (Figure 4D). While the more proliferative phenotype in SA/SA CD8^+^ T cell was similar to that reported in TSC2^–/–^ CD8^+^ T cells, TSC2^–/–^ CD8^+^ T cells were also prone to become terminally differentiated (10) in contrast to the SA/SA phenotype. In a 3-way coadoptive comparison of WT/SA OTI CD8^+^ T cells with WT and SA/SA T cells introduced to the same WT host, the WT/SA cells displayed greater proliferation over WT at nearly the same level as in SA/SA cells (Figure 4E). Thus, a single copy of the SA mutation also promoted greater T cell proliferative responses in vivo. There were also more SA/SA CD8^+^ T cells at later time points (6 weeks) after infection when memory formation has taken place, indicating that they retained their ability to persist long-term in striking contrast to TSC2^–/–^ CD8^+^ T cells (Figure 1D and Figure 4F).

To test if SA mutated CD8^+^ T cells formed bona fide memory T cells unlike TSC2^–/–^ CD8^+^ T cells based on their recall capacity on a per cell basis (10), we repeated the coadoptive transfer study using resting memory WT and SA/SA CD8^+^ T cells extracted after 90 days, sorting to equalize the number of cells for each genotype, and retransferring into naive hosts that were then infected with LM-OVA (Figure 4G). To provide a fair analysis of 1:1 recall potential, we confirmed there was no difference in memory cell phenotype (e.g., in their CD62L^+^KLRG1^–^ expression levels) prior to LM-OVA rechallenge (Figure 4G, lower). On day 5 after rechallenge, there were far more SA/SA compared with WT CD8^+^ T cells (Figure 4H), indicating the former’s ability to mount a strong memory recall, a key hallmark of memory T cells. Similar results were obtained in studies using WT/SA CD8^+^ T cells and LCMV Armstrong as the acute infection model (Supplemental Figure 5, B–D). Thus, the SA mutation at S1365 enhances CD8^+^ T cell effector generation without compromising their memory formation or recall capacity in response to reinfection in vivo, both differing significantly from the TSC2^–/–^ CD8^+^ T cell phenotype.

### TSC2 SA T cells display enhanced activation under conditions of cellular stress.

**Figure 5:**
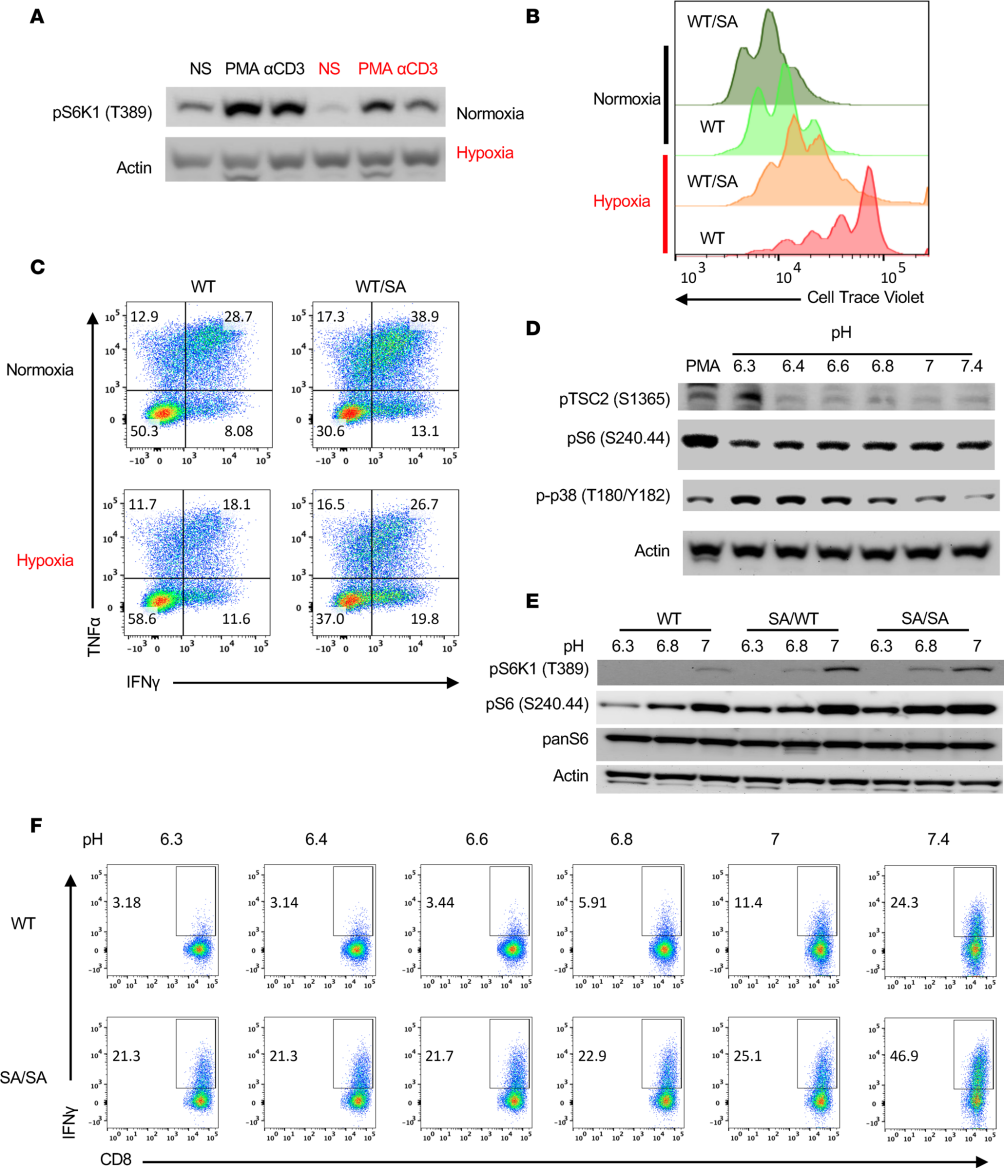
SA mutation in CD8^+^ T cells amplifies mTORC1 activation under cellular stress. (**A**) Resting CD8^+^ T cells stimulated with PMA or TCR (αCD3) in normoxic or hypoxic (2% O_2_) conditions for 15 minutes and assayed by immunoblot for mTORC1 activity via pS6K1. (**B**) Proliferation analysis of stimulated OTI CD8^+^ T cells with WT or WT/SA TSC2 in normoxia or hypoxia. Cells expressing WT/SA proliferate more in both conditions. (**C**) Flow cytometry analysis for cytokine function from IL-2 generated cytotoxic WT versus TSC2-SA mutant CD8^+^ T cells that were rechallenged overnight in normoxic or hypoxic (2% O_2_) conditions. (**D**) Immumoblot for phosphorylated TSC2-S1365, S6, and p38 MAP kinase from IL-2 pretreated CD8^+^ T cells and then cultured in neutral or more acidic media. (**E**) Activated WT, mutant TSC2 WT/SA and SA/SA CD8^+^ T cells were exposed to various media at various pH for 90 minutes and assayed by immunoblot analysis for mTORC1 activity. (**F**) Effector TSC2 WT or TSC2 SA/SA CD8^+^ T cells were stimulated with PMA and ionomycin in various pH level media to assess IFN-γ via flow cytometry. Data are representative of at least 3 independent experiments, except **E** and **F**, with 2.

The tumor microenvironment is often hypoxic and acidic that hampers mTORC1 activity and antitumor immune responses (23). Since TSC2 is a central metabolic and stress hub in mammalian cells, we hypothesized that the SA mutation in CD8^+^ T cells might provide an intrinsic advantage to support better proliferation and effector function under such environmental stress. With respect to hypoxia, mTORC1 activation (pS6K1) by either acute PMA or TCR stimulation was repressed at 1%–2% O_2_ compared with normoxic conditions (Figure 5A). When naive WT or WT/SA CD8^+^ T cells were stimulated under either condition, proliferation was greater in WT/SA CD8^+^ T cells in both normoxic and hypoxic conditions (Figure 5B), and effector function was greater upon rechallenge (Figure 5C) in both as well. Acidosis also depresses mTORC1 activation, and this is accompanied by greater p38 and TSC2 S1365 phosphorylation in mouse (Figure 5D) and human T cells (Supplemental Figure 6). If the rise in TSC2 pS1365 was a mechanism to suppress mTORC1 at low pH, one would anticipate T cells expressing the SA mutation to enhance mTORC1 despite acidosis (Figure 5, E and F). Greater mTORC1 activity was observed in CD8^+^ T cells expressing either WT/SA or SA/SA mutant TSC2 (Figure 5E), and increased IFN-γ production was present in in SA/SA versus WT CD8^+^ T cells over a broad range of pH (Figure 5F). These findings show that S1365 is a potent signaling node that responds in stressful environmental conditions and that its inhibition by the SA mutation enhances CD8^+^ T cell mTORC1 activation and effector function despite hypoxia or acidosis.

### TSC2 SA mutation enhances antitumor adoptive cellular therapy.

**Figure 6:**
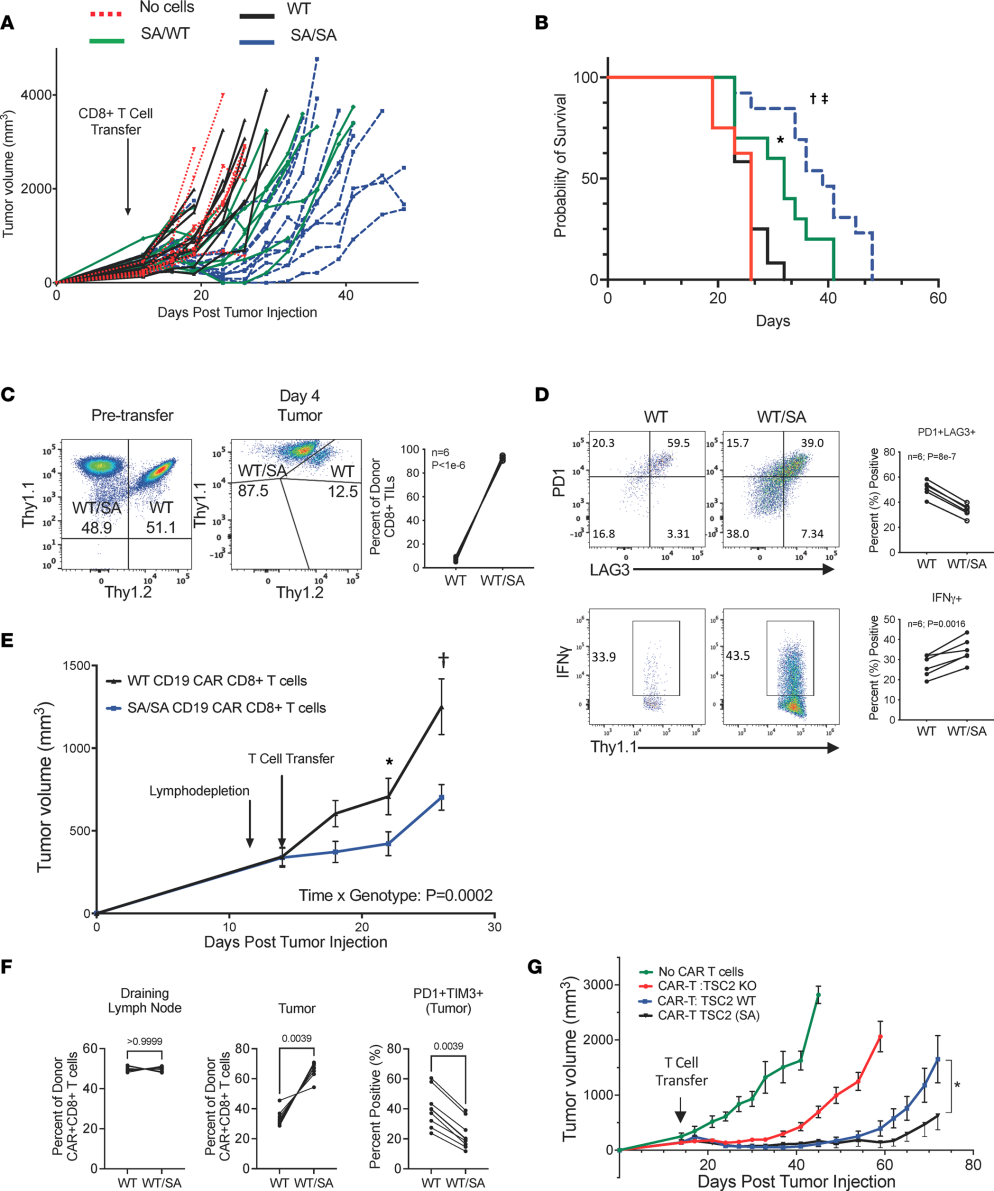
TSC2 SA CD8^+^ T cells promote strong anti-tumor immunity with adoptive T cell therapy. (**A**) WT mice with B16-melanoma OVA cells received preactivated WT (*n* = 12), TSC2 WT/SA (*n* = 10) or SA/SA (*n* = 13) OTI CD8^+^ T cells (or no cell controls, *n* = 8). Tumor volume was assessed every 2-3 days. (**B**) Survival curves with Mantel-Cox test for differences. Mice receiving TSC2 WT/SA and SA/SA CD8^+^ T cells had better survival over TSC2-WT (P=0.0085 and 1.7e-5, respectively) with some dose dependence (WT/SA versus SA/SA P=0.027). (**C**) *Left:* Equal number of activated WT and TSC2 (SA) heterozygous OTI^+^ CD8^+^ T cells co-transferred to same B16-melanoma OVA bearing host. *Middle:* Relative counts of TSC2 WT or WT/SA CD8^+^ T cells by flow cytometry in tumor. *Right:* summary data; P values for paired t-test. (**D**) Exhaustion profile (top) and function (bottom) of donor TSC2 WT or WT/SA CD8^+^ from these tumors. Quadrant numbers are percent cells in each; summary on right, paired T-test. (**E**) Murine CD19 CAR-T model targeting B16 tumors expressing human CD19. Growth curves until first sacrifice (WT *n* = 15 and SA/SA *n* = 16). Interaction of time and TSC2 genotype determined by 2W repeated measures ANOVA. Sidak’s multiple comparisons test: *P=0.07, †P=0.00002 between curves. (**F**) Similar experiment as in 6E, CAR-T cells tumor (day 8) were majority WT/SA displaying less exhaustion. WT and WT/SA CAR-T cells similar in draining lymph node. Wilcoxon, *n* = 9-10. (**G**) CAR-T experiment using human CD70 CAR-T cells against CD70 expressing human small cell lung tumor (NCI-H1975) in NSG mice (No cells *n* = 4, control *n* = 12, TSC2KO *n* = 12, TSC2 (SA) *n* = 12). *P=0.00006 for interaction of time and TSC2 genotype comparing TSC2 WT to SA. Data are representative of at least 2 independent experiments.

Having established that TSC2 S1365 regulates CD8^+^ T cell effector and memory generation as well as CD8^+^ T cell responses under hypoxic and acidic conditions, we hypothesized that CD8^+^ T cells harboring the TSC2 SA mutation would demonstrate superior antitumor activity when used in adoptive cell therapy. We first tested the B16-OVA model, which is known to be relatively resistant to adoptive cellular therapy. Indeed, transfer of activated WT OTI CD8^+^ T cells had a negligible effect on tumor growth or long-term survival, whereas transfer of either WT/SA or SA/SA OTI CD8^+^ T cells significantly inhibited both tumor growth (Figure 6A) and prolonged survival (Figure 6B), the latter with a significant dose response effect (survival log-rank Mantel-Cox; *P* = 0.0085 for WT versus WT/SA cells, *P* = 0.00002 for WT versus SA/SA cells, and *P* = 0.027 between WT/SA and SA/SA cells). Given the phenotype with WT/SA CD8^+^ T cells and greater ease in their generation, we then tested how these T cells responded in the tumor microenvironment compared with WT counterparts. Equal numbers of activated WT/SA and WT OTI CD8^+^ T cells were coadoptively transferred into B16-OVA tumor–bearing mice. Four days later, before tumors regressed, tumors were excised, and the relative percentage of WT and WT/SA donor CD8^+^ T cells in the tumor was determined. The vast majority (~90%) of donor TILs were mutant WT/SA CD8^+^ T cells (Figure 6C). WT/SA CD8^+^ T cells were also phenotypically less exhausted than WT cells based on lower surface expression levels of the exhaustion markers PD1 and LAG3 with enhanced cytokine IFN-γ expression (Figure 6D).

We next tested if the SA mutation enhanced the efficacy of adoptively transferred CD8^+^ T cells using a murine CD19 CAR-T cell model with WT or SA/SA CD8^+^ CAR-T cells (Supplemental Figure 7A) (24). The SA/SA CD19 CAR-T cells also significantly suppressed tumor growth (Figure 6E). The analogous coadoptive transfer with equal numbers of WT and WT/SA CD19 CAR-CD8^+^ T again found far more WT/SA CAR-T cells in the tumor, whereas their abundance in the draining lymph node was similar to their abundance in WT CAR-T cells. This is consistent with a survival advantage for WT/SA CAR-T cells in the tumor microenvironment (Figure 6F and Supplemental Figure 7, B and C).

Since all of these models involved mouse T cells, we wanted to test whether engineering this mutation in primary human T cells would yield similar results to suppress tumor growth with adoptive cell therapy. Human TSC2^–/–^ CD70 CAR-T cells were first generated and then, in a subgroup, further modified to express the TSC2 S1364A mutation (Supplemental Figure 8). The antitumor efficacy of these T cells was further compared with WT control CAR-T cells or no cells into NSG mice bearing CD70-expressing human non–small cell lung cancer (NSCLC) tumors. Mice receiving SA CAR-T cells displayed slower tumor growth for a longer duration as compared with mice receiving WT CAR-T cells and TSC2^–/–^ CD70 CAR-T cells (Figure 6G). This 3-way comparison was reproduced using a lower dose of CAR-T cells (Supplemental Figure 9A). In vitro, SA CAR-T cells had greater IFN-γ and TNF-α production compared with WT T cells under a wide range of pH conditions (Supplemental Figure 9B). Interestingly, while all 3 CAR-T cell groups initially suppressed tumor growth, the TSC2^–/–^ were the least effective in controlling tumor growth over time. This is very much consistent with the loss of memory potential in TSC2^–/–^ T cells and provides a striking example of how mutating a single amino acid (S1365A) on TSC2 substantially differs from deleting TSC2 entirely.

## Discussion

MTOR has emerged as an important integrator of signals dictating T cell differentiation and function. A critical component of its ability to regulate immune cells is via metabolism (9), as mTORC1 activation promotes T cell effector function by upregulating glycolytic metabolism (10, 25). Likewise, inhibition of mTORC1 enhances long-lived memory CD8^+^ T cell generation by inhibiting glycolytic programs and promoting oxidative phosphorylation. In addition, the role of mTOR in regulating cytokine signaling and the expression of canonical transcription factors have been shown to contribute to the ability of signaling downstream of mTOR to regulate T cell differentiation and function (26). It is this complex coordination of T cell functionality that has made manipulation of mTORC1 therapeutically challenging. mTORC1 inhibitors such as rapamycin or suppressors of upstream activation by PI3K and Akt have long been a target for cancer, but they also suppress T cell immune effector function. Here, we have shown that a single mutation on TSC2 substituting alanine for serine at residue 1365 to block its intrinsic phosphorylation increases mTORC1 activation upon T cell stimulation, leading to enhanced effector differentiation and function. Importantly, mutation at this site does not result in constitutive mTORC1 activation but selectively enhances activity upon stimulation. For CD8^+^ T cells, this translates to the ability of the SA mutation to promote the generation of more effector T cells with a concomitant increase in long-lived memory T cells. When interposed with adoptive T cell therapy (e.g., CAR-T), this mutation enhances tumor growth control and overall survival, with signs of better T cell persistence with less exhaustion. This constellation of features is keenly sought after to improve CAR-T and other adoptive cell therapies, and the present findings support use of gene editing to generate a TSC2 SA mutation as part of the strategy.

While mTORC1 is regulated by a multiplicity of signals, including glucose, lipids, amino acids, and signaling kinases, it is the latter that prominently converge on TSC2 to modify mTORC1 complex signaling (13, 14, 16, 27, 28). Akt, ERK1/2, p38, RSK1 kinase activation, and 14-3-3 binding all relieve TSC2 inhibition of mTORC1, whereas AMP-activated kinase and GSK-3β enhance TSC2-mediated mTORC1 suppression. Despite this central role, modification of TSC2 has previously been difficult to leverage as a means of selectively manipulating mTORC1 activity. First, most kinase-regulated changes engage multiple residues to generate the effect, making gene editing more complex. Second, mutagenesis of these sites to prevent or mimic phosphorylation has led to altered constitutive mTORC1 activity. S1365 on TSC2 is unique in this regard, since we find minimal basal impact with either modification yet a very potent effect upon mTORC1 stimulation. This was first revealed in cardiac myocytes and fibroblasts (18) and here is shown to be recapitulated in T cells. It is this feature that differentiates the SA mutant from TSC2^–/–^ T cells, the latter also generating strong effector differentiation and function at the expense of poor long-term persistence and memory formation. The SA mutation, by contrast, preserves overall TSC2 functionality and amplifies effector function and proliferation, but it only does so with selective stimulation conditions, which preserves long-term persistence and memory formation when the infection has cleared.

In contrast to cardiac myocytes, phosphorylation of TSC2 S1365 in T cells is not mediated by cyclic GMP stimulated kinase (cGK-1) but rather by p38 MAPK. PKC can also achieve this but via a p38 downstream mechanism. Importantly p38 and PKC are both activated upon TCR stimulation along with ERK1/2 and Akt. While concordance of p38 and TSC2 S1365 phosphorylation in CD8^+^ T cells was observed with several different interventions (e.g., TCR and PMA stimulation, acidosis, and H_2_O_2_), net mTORC1 activity varied between them, rising with TCR and PMA, declining with acidosis, and remaining unchanged with H_2_O_2_. Thus, a rise in pS1365 does not guarantee what will transpire in net mTORC1 activity. What is consistent is that, by suppressing pS1365 with the SA mutation, mTORC1 activity rises with these stimuli. This supports S1365 as a coupled negative regulator rather than primary determinant of mTORC1 activity. While not explored in detail here, our prior study found S1365 phosphorylated along with MAPK but not Akt activation, and this predicted whether the SA (or SE) phosphomutants would alter mTORC1 costimulation (19). Here, we found analogous disparate effects on TSC2 S1365 phosphorylation arising from TCR versus IL-2 stimuli, and we suspect S1365 modifications will have analogous selectivity to T cell input signaling and, thus, cytokine control. Importantly, TCR-induced mTORC1 activity regulated by the S1365 status provides a means to improve CAR-T cell activation and function in response to tumor antigens.

Tumor-infiltrating lymphocytes in different tumor models showed substantial bias to CD8^+^ T cells harboring the SA mutation over WT T cells. This could reflect metabolic changes and/or synthetic changes coupled to mTORC1 activation that enabled TSC2 SA T cells to better survive and proliferate within the hostile tumor microenvironment. Our in vitro data showing both enhanced proliferation and cytokine generation of such SA T cells despite hypoxia or acidosis are consistent with this. Expression of the SA mutation in the KI mouse has also been shown to enhance cardiac protection against ischemic damage by a mechanism linked to greater glucose versus fatty acid metabolism (20). Here, we found SA–T cells also consistently exhibit less markers of exhaustion, supporting an underlying capacity to regulate mTORC1 activation dependent on the level of stressors and activation.

There are some limitations to the present study. Given the TSC2 S1365A–KI mouse was a global mutation, we did not test various immune responses to virus or tumors in these animals themselves but used TCR transgenic models for focused T cell analysis, which enabled us to track T cells throughout the immune response process. We considered studies that assessed the endogenous immune response with our global KI mice, but since all immune cells (indeed all cells) contained the same SA mutation, we felt they would not allow us to determine what cells were responsible for any observations and conclusions. OVA is an artificial antigen and elicits stronger immune responses than native ones. However, for the purpose of establishing signaling mechanisms and T cell phenotype and fate, this approach was most direct. For the tumor therapy applications, we also used physiological antigens (e.g., CD19, CD70) incorporated into CAR-T adoptive therapies, and the results were similar in nature to what we observed with the OVA-dependent models. Lastly, while not every assay had more than 2–3 biological replicates, there was substantial concordance of findings among different independent experiments that strengthen the main conclusions of the work.

The current study findings have potential clinical implications. Immunotherapy in the form of adoptive cell therapy has emerged as a potent means to treat cancer (29). ACT provided by CAR-T cells is an FDA-approved treatment for several hematologic malignancies. However, there remain many challenges, including the lack of long-lived memory of the in vitro engineered CAR-T cells and less success in the treatment of solid tumors (30). This may be due in part to the hostile tumor microenvironment, and to that end, creating CAR-T cells with a TSC2 SA mutation may help circumvent this limitation by improving effector function and long-term survival even in hypoxic and/or acidic microenvironments. Furthermore, use of these modified T cells may promote better persistence of CAR-T cells that can be quickly reactivated and expanded to prevent relapse. This also suggests potential synergy with checkpoint inhibitors, as these therapies can reawaken quiescent T cells but not those that are terminally exhausted. Finally, while the technology to create such genetically engineered CAR-T is now available, the fact that the TSC2 SA mutation acts as an autosomal dominant mutation, with a generally similar impact from a hetero- and homozygote mutation, suggests that mutation efficiency of even 50% should be quite therapeutically effective. Since engineering of CAR-T cells becomes more diverse and facile, targeted base editing or even expression of a mutant protein possibly linked to a controllable inducer are 2 such examples of exploiting our findings clinically. These and further translational studies to test the impact of TSC2 SA should define their value for immuno-oncology therapies.

## Methods

### Mice.

Six- to 10-week-old male or female mice were used for performing all the experiments in this study. NSG, C57BL/6J, CD4 Cre, CD8^+^ OTI (OVA TCR), CD90.1/Thy1.1^+^, and CD45.1 mice were obtained from The Jackson Laboratory, and CD4^+^ 5CC7 (PCC TCR) was obtained from Taconic and bred in-house. P14 (gp33 TCR) were provided by David A. Hildeman, University of Cincinnati (Cincinnati, Ohio, USA). Mice with knock-in mutations at S1365 (SA and SE) were as first generated on a C57BL/6J background (18) and used in the study. Mice with loxP flanked TSC2 alleles were generated by the laboratory of Michael Gambello (University of Texas Health Science Center at Houston, Houston, Texas, USA) and bred to CD4 Cre. Genotyping was determined by respective protocols. No empirical test was performed for choosing sample size prior to experiments. No randomization of samples or animals was used, nor were investigators blinded throughout the study.

### Antibodies and reagents.

Class-I peptide (SIINFEKL, OVA I) was purchased from AnaSpec. Fc Block (2.4G2) and stimulatory in vivo plus grade αCD3 (2C11) and αCD28 (37.51) were purchased from BioXcell. Cell Proliferation Dye-eFluor450 was purchased from eBiosciences. Rapamycin was purchased from LC Laboratories. All small-molecule inhibitors were purchased from Cayman Chemicals and used at the following concentrations: ERK (U-0126, 10 μM), PKC (Calphostin C, 1 μM), p38 (SB 203580, 5 μM), and mTORC1 (Rapamycin, 1 μM). Secondary fluorophore conjugated antibodies were purchased from Invitrogen: anti–rabbit Alexa Fluor 647 (Thermo, A-31573). IL-2 (10 ng/mL) and IL-7 (10 ng/mL) were purchased from Peprotech. PMA (50ng/mL), ionomycin (100ng/mL), H_2_O_2_ was purchased from MilliporeSigma. Golgi Stop and Plug were purchased from BD Biosciences. Sources, clone, and catalog number for these reagents as well as for all antibodies used are provided in Table 1.

### Radiolabeled ^33^P TSC2 kinase assay.

TSC2 KO HEK cells were transduced with adFLAG-TSC2. Samples were collected and underwent immunoprecipitation for FLAG. The kinase assay was performed while TSC2 was in the immunocomplex on the magnetic beads, with either TSC2 alone (negative control), activated ERK2 kinase + TSC2 (positive control), p38 alone (negative control), or increasing p38 dose combined with TSC2. Samples were then eluted from the beads by boiling in sample loading buffer and subjected to SDS-PAGE and Western blotting for TSC2 and FLAG and imaging by film for ^33^P incorporation.

### Activating T cells in vitro.

Naive resting splenocytes from spleens and lymph nodes were combined for all experiments. In summary, single-cell suspensions were created by mashing organs through a 70 μM filter. Splenocytes from WT, TSC2 KO, and TSC2 SA mice were then stimulated with soluble αCD3 (3 μg/mL) or isolated CD8^+^ T cells (Negative Selection, BioLegend) with plate-bound αCD3 (5 μg/mL) and soluble αCD28 (2 μg/mL) for 48 hours being being expanded in IL-2 (10 ng/mL) for 4–5 days to generate resting previously activated CD8^+^ T cells. WT or TSC2 mutant (SA or SE) OTI CD8^+^ T cells (final 5 × 10^6^/mL) were stimulated with OVA I peptide (100 ng/mL, SIINFEKL, Anaspec) for 48 hours and were then expanded and fed with fresh media and IL-2 (10 ng/mL) daily for 4–5 days to generate resting previously activated effector CD8^+^ T cells for further functional or signaling assays. 5CC7 CD4^+^ PCC transgenic T cells were stimulated with 5 μg/mL PCC peptide for 48 hours and were then expanded in IL-2 (10 ng/mL) for 4–5 days to generate previously activated CD4^+^ T cells for some signaling analysis. Cell proliferation of OTI CD8^+^ T cells with peptide (100 ng/mL) stimulation was monitored with cell trace violet (CTV, eBio) between 48 and 72 hours.

### T cell activation or stress-induced signaling.

Naive T cells from spleens and lymph nodes were combined for all experiments. In summary, single-cell suspensions were created by mashing organs through a 70 μM filter, and CD8^+^ T cells were isolated using negative selection isolation. Naive or resting IL-2 expanded T cells from WT, TSC2-KO (T-TSC2^–/–^), mutant TSC2 SA, and mutant TSC2 SE mice were stimulated with cross-linked Armenian hamster IgG, αCD3 (3 μg/mL), αCD28 (2 μg/mL), PMA, H_2_0_2_, or other activation stimulus at indicated time points for flash freezing to assess signaling via immunoblotting. Primary T cell cultures were maintained in RPMI-1640 media (Corning 10-040-CV) with 10% FBS (Gemini Bioproducts), 2 mM L-glutamine (Corning), 10 mM HEPES (Corning 25-060-CI), gentamycin (50 μg/mL, Quality Biological), nonessential amino acids (100×, Thermo Fisher Scientific), and β-mercaptoethanol (50 μM, MilliporeSigma) in standard humidified 5% CO_2_, 37°C tissue culture incubators. Experimental culture conditions are described below.

### Assessing T cell mTOR activity via flow cytometry.

Primary splenocytes were derived and stimulated as detailed above. Following stimulation, splenocytes were fixed with 2% PFA for 10 minutes at 37°C before being washed 2 times with PBS. Cells were permeabilized with ice-cold 90% methanol for 20 minutes at –20°C. Cells were washed 3 times with 1% FBS/PBS (staining solution). Next, cells were stained with αCD4 (1:500), αCD8 (1:500), and anti p-S6 (S240.44) (1:2,000) in staining solution for 45 minutes at room temperature. Cells were then washed 2 times with staining solution before stained with Rabbit IgG AF647 (1:500, Thermo, A-31573) for 30 minutes at room temperature. Cells were then washed 2 times afterward. Gates were set appropriately with the aid of unstimulated and secondary-alone controls. All experiments were performed on a BD FACS Calibur, LSR II, or Aria II and analyzed using FlowJo software analysis.

### Intracellular cytokine stimulation.

Viable T cells were enriched with ficoll gradient and were then stimulated with PMA/ionomycin (4–5 hours) with Golgi Stop or platebound αCD3 (1 μg/mL) and soluble αCD28 (2 μg/mL) (14–16 hours) with Golgi Plug to assess cytokine production. Cells were collected and then stained with viability dye and surface staining for 20 minutes at 4°C. Next, cells were washed and fixed with 100 μL of BD CytoFix/Perm kit for 30 minutes at room temperature, washed with BD 1× PermWash buffer, and stained with intracellular cytokine staining in 1× BD PermWash for 45 minutes at room temperature before being analyzed on a flow cytometer (BD Celesta).

### Low pH experiments.

For experimental manipulation of pH, CD8^+^T cells from WT, TSC2-KO, TSC2 SA were stimulated in RPMI-1640 (MilliporeSigma, R1383 with 11.1 mM glucose restored) supplemented with 10% FBS, 2 mM L-glutamine, gentamycin (50 μg/mL), and β-mercaptoethanol (50 μM) in which the bicarbonate-CO_2_ buffering was replaced with 25 mM PIPES and 25 mM HEPES in atmospheric CO_2_ as above. Cultures were maintained at 37°C in a humidified incubator. When prepared, slightly concentrated media were split into multiple volumes before adjusting pH to target values, and sterilizing by filtering, ensuring identical media composition in all regards other than pH. pH of stored media was frequently monitored to guard against slow drift and to ensure correct record of experimental conditions (31).

### LCMV, vaccinia-OVA, and LM-OVA CD8^+^ T cell adoptive transfer model.

For assessing acute and memory responses in vivo, different congenically marked isolated CD8^+^ OTI or P14 T cells were mixed at equal ratios (1 × 10^3^ to 5 × 10^3^ cells of each population) and coadoptively transferred into WT hosts. Recipient mice were shortly later infected with double attenuated listeria monocytogenes (LM) (OVA or gp33) (5 × 10^6^ cfu/mouse, i.v.) or LCMV Armstrong (2 × 10^5^ pfu/mouse, i.p.). LCMV Armstrong was provided by Susan Kaech (Salk Institute, San Diego, CA, USA). Spleens were collected 4–8 days after infection. Blood and spleens were collected at indicated time points. For the secondary adoptive transfer experiment, resting memory splenocytes were harvested after naive adoptive transfer and infection. Cells were sorted for equal donor CD8^+^ T cells and again coadoptively transferred into naive hosts for secondary recall with pathogen.

### B16-OVA and B16-CD19 adoptive T cell therapy model.

For the B16 adoptive T cell therapy models, naive C57BL/6J WT mice received a s.c. injection of 2.5 × 10^5^ B16-OVA melanoma cells (gift of Hyam Levitsky, Century Therapeutics, Seattle, Washington, USA) cultured in vitro under OVA selection media containing 400 μg/mL G418 (Invitrogen) or 5 × 10^5^ B16-OVA-CD19 (provided by Anjana Rao [La Jolla Institute for Immunology, San Diego, CA, USA]; 24). In the B16-OVA model, 11 days after tumor inoculation, mice received an adoptive transfer of 7.5 × 10^5^ activated WT or mutant TSC2 SA OTI CD8^+^ T cells derived from splenocytes, which had been stimulated in vitro with OVA I peptide (100 ng/mL) for 48 hours and expanded in IL-2 (10 ng/mL) for an additional 48 hours. On day 4 after activation, cells were subjected to ficoll gradient to enrich for viable CD8^+^ T cells. TSC2 WT or SA mutant CD8^+^ OTI T cells were transferred into tumor-bearing mice for tumor outgrowth experiments. Mice were randomized into groups on the day of therapy. In the B16-OVA-CD19 model, mice were lymphodepleted with 200 μg cyclophosphamide (i.p., MilliporeSigma) 1 day before receiving CD19 CAR^+^ CD8^+^ T cells. Tumor volume was calculated using the formula for the prolate ellipsoid, (L × W^2^)/2, where L represents length and is the longer of the 2 measurements and W represents width. Tumor burden was assessed every 2–4 days by measuring length and width of tumor. Mice were sacrificed when tumors exceeded 2 × 2 cm, when tumors were necrotic, or when mice experienced visible signs of discomfort. To analyze tumor infiltrating T cells (TILs), a 1:1 ratio of cells was mixed to analyze infiltrate of CD8^+^ T cells on days 4–8 after transfer.

### Retroviral transduction of CD19 CAR CD8^+^ T cells.

In brief, polyclonal isolated WT or mutant TSC2 SA CD8^+^ T cells were stimulated for 24 hours with plate-bound αCD3 (5 μg/mL) and soluble αCD28 (2 μg/mL) with human IL-2 (100 units/mL). CD19 CAR^+^ retroviral transductions were performed in 6-well non–tissue-treated coated plates with 20 μg/mL retronectin (Takeda). Fresh virus of (MSCV-myc-CAR-2A-Thy1.1, Anjana Rao, Addgene, 127890) containing chimeric antigen receptor (CAR) was made using Platinum-E (Plat-E) Retroviral Packaging Cell Line (Cell Biolabs). Fresh virus with human IL-2 was spinfected onto retronectin-coated plates according to protocol (no. 33156338). After spinfection of virus, activated T cells were slowly layered on top of the virus and briefly spun for 2 additional days on virus-coated plates for viral transduction. On day 3, T cells were collected and expanded in fresh media with 100 units of human IL-2/mL for 4 additional days before transferring into B16-OVA-CD19 tumor-bearing hosts. CD19 CAR efficiency was assessed by Thy1.1 surface staining. Equal CAR^+^CD8^+^ T cells were transferred into tumor-bearing mice by normalizing with flow cytometry for single transfer (efficacy) and coadoptive experiments (TILs).

### TIL harvest.

Four days after CD8^+^ OTI or CD19 CAR-T cell transfer, tumors were harvested from mice and digested in 2 mg/mL collagenase I (Invitrogen) with DNase I (Roche) in RPMI 1640 supplemented with 2% FBS. Tumors were digested with constant rotation at 37°C for 30 minutes, followed by quenching with EDTA. Cells were then filtered and processed into single-cell suspensions. Cells were then stained with antibodies for subsequent flow analysis of transferred CD8^+^ T cells defined by congenic markers from tumor-bearing hosts.

### Immunoblot analysis.

For immunoblot analysis, T cells were harvested by centrifugation (300 *g* at room temperature for 7 minutes) and resuspended in ice-cold lysis buffer (20 mM Tris [pH 7.5], 150 mM NaCl, 1 mM EDTA, 1 mM EGTA, 1% Triton X-100, 2.5 mM sodium pyrophosphate, 1 mM β-glycerolphosphate [glycerol-2-phosphate], 1 mM sodium orthovanadate, 1 mM PMSF, 1× protease inhibitors; Roche) and lysed at 4°C for 30 minutes. Lysates were cleared of debris by high-speed centrifugation (15,000 *g* at 4°C for 15 minutes). Equal protein mass from each condition was mixed with 4× LDS buffer (Invitrogen) and boiled for 10 minutes. Lysates were then loaded into NuPAGE gels (4%–12% Bis–Tris gels, Invitrogen) and run at 150 V for 90 minutes. Protein was transferred to polyvinylidene fluoride (PVDF) membranes with transfer buffer (1× NuPAGE Transfer Buffer [Invitrogen] with 20% methanol) at 30 V for 90 minutes. Membranes were blocked in 5% nonfat dry milk (NFDM) for 60 minutes, washed briefly with Tris-buffered saline + 0.1% Tween-20 (TBST), and probed with primary antibody in 4% NFDM in TBST overnight at 4°C. Membranes were washed with TBST 3 times for 10 minutes and probed with secondary antibody conjugated to HRP in NFDM. Membranes were washed 2 times in TBST for 5 minutes and then once in Tris-buffered saline once for 5 minutes. Enhanced chemiluminescent picoplus substrate (Thermo Fisher Scientific) was used to detect HRP-labeled antibodies. Blots were developed using a Biospectrum Multispectrum Imaging System, and images were acquired and analyzed using VisionWorks, LS Image Acquisition, and Analysis software (UVP).

### Human CD70 CAR-T cells.

Western Blot assay was performed with normalized protein concentrations of CAR-T cell pellet lysates (Lysis Buffer R0278, Sigma-Aldrich) in the presence of Halt Protease and Phosphatase Inhibitor (Thermo Fisher Scientific, 78442). Equal total protein concentration was assayed using WES (Bio-Techne), with detection of TSC2 (Cell Signalling Technology, Tuberin/TSC2, D93F12, XP Rabbit mAb) and GAPDH (14C10, Cell Signaling Technology, 2118). TSC2 expression was normalized to GAPDH via signal peak area.

### CRISPR/Cas9 RNP system of naive T cells.

Modified single-guide RNAs (sgRNAs) were designed and synthesized by Synthego. Ribonucleoproteins (RNPs) were prepared by incubating sgRNAs and Cas9 nuclease (Integrated DNA Technologies, IDT) for 10 minutes at room temperature. For the delivery of RNPs, isolated WT P14 CD8^+^ T cells were washed with PBS and mixed with RNPs by using P3 Primary Cell 4D-NucleofectorTM X Kit (Lonza) immediately prior to electroporation (Lonza 4D-nucleofactorTM core unit, program DN100). Electroporated T cells were recovered and washed with T cell culture media. Cells were activated with plate-bound αCD3 and soluble αCD28 and were expanded in IL-2 for subsequent immunoblot analysis. The sequences of sgRNAs are as follows:

Ctrl: 5′ - GCACUACCAGAGCUAACUCA - 3′ ; TSC2-g1: 5′ - AGCAUGCAGUGGAGGCACUU - 3′; TSC-g2: 5′ - UUUGUCAUGGCAGCAUGCAG - 3′; p38a-g1: 5′ - GUACCUGGUGACCCAUCUCA - 3′.

### Human CAR-T TSC2 SA/SA generation and protocol.

Guide RNA was designed to disrupt the TSC-2 gene near S1363 using homologous recombination to change serine to an alanine. Point mutations were also introduced to the donor template to stop the Cas9/guide complex from cutting the donor template and mutated gene. Homology arms were designed to the sequences flanking the mutated sequence to direct the homology directed repair.

WT sequence, GTTGGCAGG GGCATCCCCATCGAGCGAGTCGTCTCCTCGGAGGGT GGCCGG; donor template, GTTGGCAGG GGCATtCCaATtGAaCGgGTtGTggctTCtGAaGGT GGCCGG; WT sequence, GIPIERVVSSEG; and donor template, GIPIERVVASEG. The guide used was Spacer TSC-4 CATCGAGCGAGTCGTCTCCTCGG. Guide sequence was mC*mA*mU*CGAGCGAGUCGUCUCCUguuuuagagcuagaaauagcaaguuaaaauaaggcuaguccguuaucaacuugaaaaaguggcaccgagucggugcmU*mU*mU*U. mX indicates 2’O-methyl (M); X* indicates 2’-ribo 3’-phosphorothioate (S), and X indicates nucleotide.

### Human CAR-T cell production and adoptive cell therapy.

CAR-T cell constructs were synthesized and cloned into an AAV6 plasmid backbone. All CAR construct included a CD8 transmembrane domain in tandem with an intracellular 4-1BB costimulatory and CD3ζ signaling domain. Gene editing and cell preparation was performed using standard techniques as described in detail elsewhere (29). Briefly, human peripheral blood mononuclear cells (PBMCs) were thawed, and the T cells were activated with conjugated CD3/CD28 agonists for 3 days in T cell media containing human serum, IL-2, and IL-7. After activation, the T cells were electroporated with Cas9 protein and sgRNAs targeting the TRAC and B2M loci or TRAC, B2M, and CD70 with or without TSC2 loci and subsequently transduced with a recombinant AAV6 vector containing donor template DNA for insertion of the CD70 CAR construct, with or without TSC2 S1364A Δ. Following electroporation and transduction, the CAR-T cells were expanded for 7 days in T cell media containing human serum, IL-2, and IL-7. These cells were frozen in Biolife solution CryoStor CS10 Freeze Media (Thermo Fisher Scientific, NC9930384) and transferred to storage in liquid nitrogen prior to use in assays. For NCI-H1975 (ATCC) tumor inoculation, 5E6cells/mouse were injected s.c. to NSG flank in 50% Matrigel/50% Media in 0.1 mL. Once tumor reached appropriate size (150 mm^3^), frozen human CD70 CAR-T cells were thawed and washed before adoptive transfer into tumor-bearing mice to monitor tumor growth over time.

### T cell assays.

T cell assays for activity, proliferation, and cytotoxicity have been described in detail elsewhere (30). Briefly, in coculture experiments, T cells were incubated with Daudi target cells at an effector/target ratio (E:T) of 0.5:1, 1:1, and 2:1 for 20 hours. Cell-free supernatants from cells were subsequently analyzed for cytokine expression using a Luminex array (Luminex Corp, FLEXMAP 3D) according to manufacturers’ instructions. Expression of surface markers were either taken at baseline or after a period of coculture and then subjected to flow cytometric analysis. Antigens were specifically stained using the following antibody clones for flow cytometry where indicated: CCR7 (CD197, BioLegend) and CD45RA (H1100, BioLegend). For proliferation, cells were counted every 1–3 days. Percent specific lysis for cell lysis determination may be calculated from live cell number, target cells prestained with specific marker (efluor670; Invitrogen eBioscience Cell Proliferation Dye eFluor 670, 50-246-095), normalized with flow cytometry counting beads (CountBright beads; Absolute Counting Beads for flow cytometry, Invitrogen, C36950).

### TIDE analysis of genome editing.

TIDE analysis was performed as follows: isolate DNA of CAR-T cell pellets with DNEasy Blood & Tissue Kit (Qiagen, 69506), perform PCR amplification with appropriate forward and reverse primers to TSC2 S1364A Δ insertion, and then submit PRC products for sequencing (Genewiz). On receipt of sequencing data, sequences were analyzed relative to the TSC2 S1364A Δ sequence, with Tsunami software, to determine the percentage of identical or aberrant sequences, insertions, and deletions present in the samples. Additional SnapGene sequence alignment was performed with Blast 2 BlastSearch to confirm alignment.

### Statistics.

All graphs were made and statistical analyses were performed using GraphPad Prism software (v.7 and 8). All individual *P* values are provided, with a value below 0.05 considered statistically significant. All paired or unpaired parametric (Student’s *t* test) or nonparametric (Wilcoxon signed-rank; paired, ranked-sum; unpaired) tests between 2 groups were 2 tailed. One- and 2-way ANOVA (parametric) or Kruskal-Wallis rank sum (nonparametric) tests were used when comparing more than two groups and are identified in the respective figure legends. Data are shown as mean ± SD.

### Study approval.

Mice were maintained and studied in accordance with protocols approval by the Johns Hopkins University IACUC (protocol MO19M71).

### Data availability.

Teh Supporting Data Values file is provided in the supplement. This provides all the numbers for all data points displayed for each figure subpanel in which it is presented. All other data are available upon reasonable request forwarded to the corresponding author.

## Author contributions

CHP, JS, DAK, and JDP designed and oversaw the study; CHP, YD, NK, XW, BLDE, MJR, LMB, and DBH performed experiments and data analysis. JW helped with mouse genotyping and colony maintenance. DAK and JDP acquired funding. CHP, DAK, and JDP wrote the manuscript.

## Supplementary Material

Supplemental data

Supporting data values

## Figures and Tables

**Table 1:**
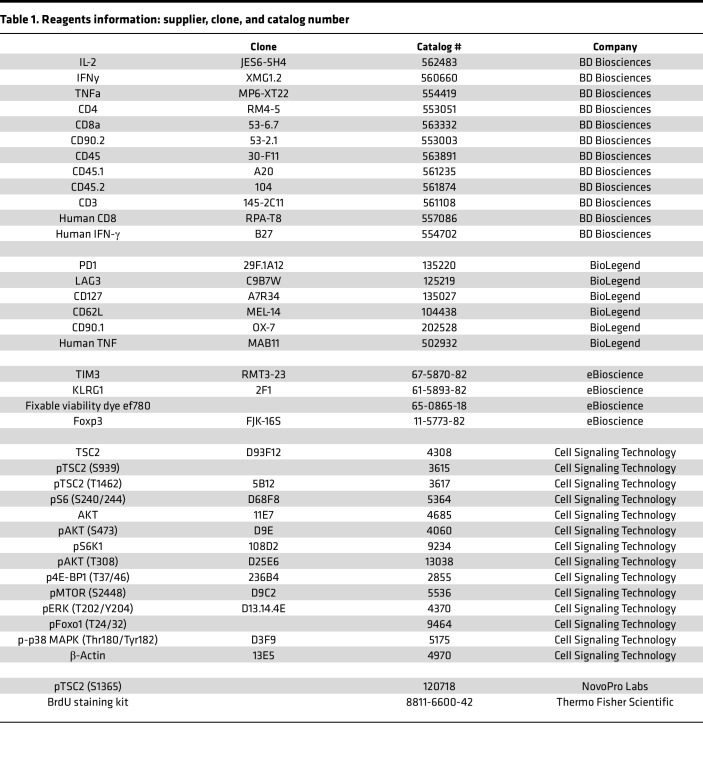
Reagents information: supplier, clone, and catalog number

## Version 1 10/03/2023

In-Press Preview

## Version 2 11/08/2023

Electronic publication
