# Supplementary material for: TSC2 S1365A mutation potently regulates CD8+ T cell function and differentiation and improves adoptive cellular cancer therapy
Source: JCI Insight. 2023 Nov 8;8(21):e167829. doi: 10.1172/jci.insight.167829 (PMC10721258; doi:10.1172/jci.insight.167829)
Supplement: Supplemental data [file jciinsight-8-167829-s180.pdf]

# Supplemental Figures and Legends

**Supplemental Figure 1**

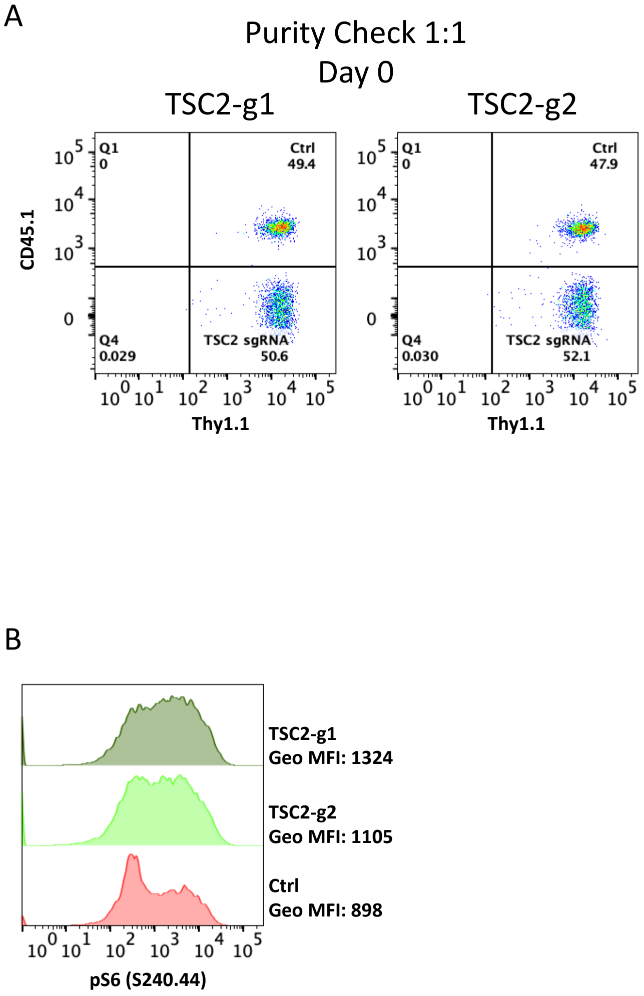


Supplemental Figure 1: **TSC2 is a central environmental hub that modulates mTORC1 activity and differentiation in T cells. A)** Control data relevant to results displayed in Figure 1D. showing by flow-cytometry that equal numbers of control (TSC2- WT, Ctrl) and TSC2 KO (by Crispr-CAS9 using either guide g1 or g2) are used as donor P14 CD8+ T cells on Day 0 of the experiment. **B)** mTORC1 activity assessed by intracellular staining of pS6 in control sgRNA and TSC2s sgRNA CD8+ T cells (e.g. TSC2-WT vs KO) in studies displayed in Figure 1. This confirms constitutive mTORC1 activation with TSC2 by either guide RNA. Experiment is representative of two independent experiments.

## Supplemental Figure 2

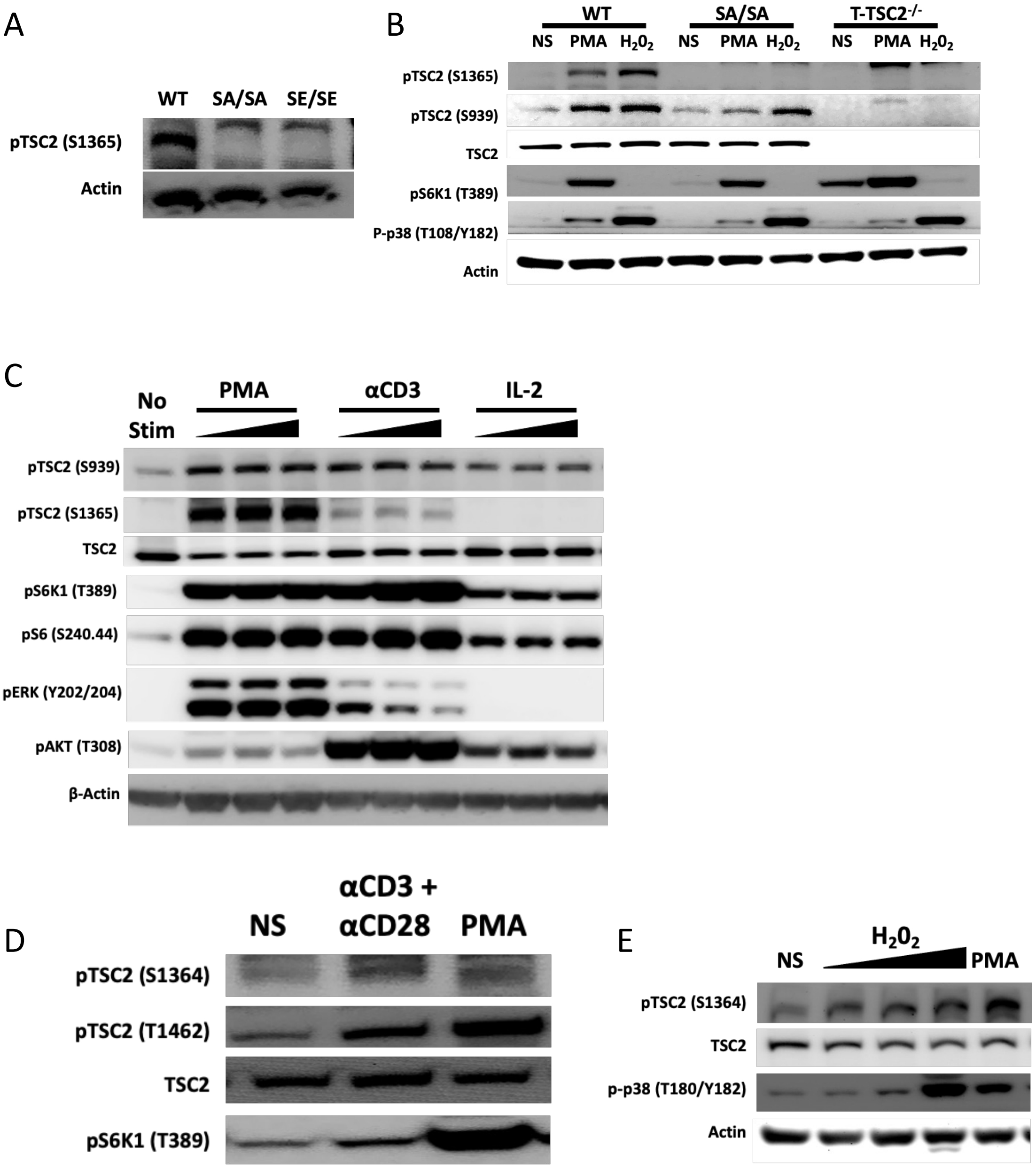

Supplemental Figure 2: **pTSC2 (S1365) is induced upon T cell activation**. **A)** Antibody generated against pS1365 shows a strong band in cells exposed to 30 minutes PMA, but this is lacking in cells expressing S1365A or S1365E mutants. **B)** Immunoblot analysis in WT, TSC2 SA/SA and T-TSC2$^{-/-}$ CD8+ T cells exposed to PMA or $H_2O_2$ stimulation (30 minutes). PMA but not $H_2O_2$ results in increased pS6K1 (mTORC1 activation) the latter associated with greater p-p38. P-S1365 is induced by both PMA and $H_2O_2$. These data are consistent with the notion that enhanced p38 activation associates with TSC2 1365 phosphorylation and reduced net mTORC1 activity. **C)** Immunoblot analysis of T cells stimulated with TCR agonism (anti-CD3, αCD3), PMA, or IL-2 at incremental doses over 30 minutes. TSC2 (S1365), TSC2 (S939; AKT site), mTORC1 activation (pS6K1 and pS6), and pAKT and pERK are assessed. Phosphorylation at TSC2 S1365 follows pERK but not pAKT changes. Data supplement those presented in Figure 1C at a single dosage of the activators but over different exposure times. **D)** Pre-activated resting human T cells were re-stimulated with TCR agonism (αCD3) and Co-Stim (αCD28) or by PMA for 30 minutes and immunoblots obtained to measure pTSC2 and pS6K1. **E)** Activated resting human T cells were exposed to various $H_2O_2$ concentrations for 30 minutes and assayed by immunoblot analysis for pTSC2 and p38 activity. Data are representative of 2 independent experiments. Data are representative of one experiment (A-B), two independent experiments (C-E).

## Supplemental Figure 3

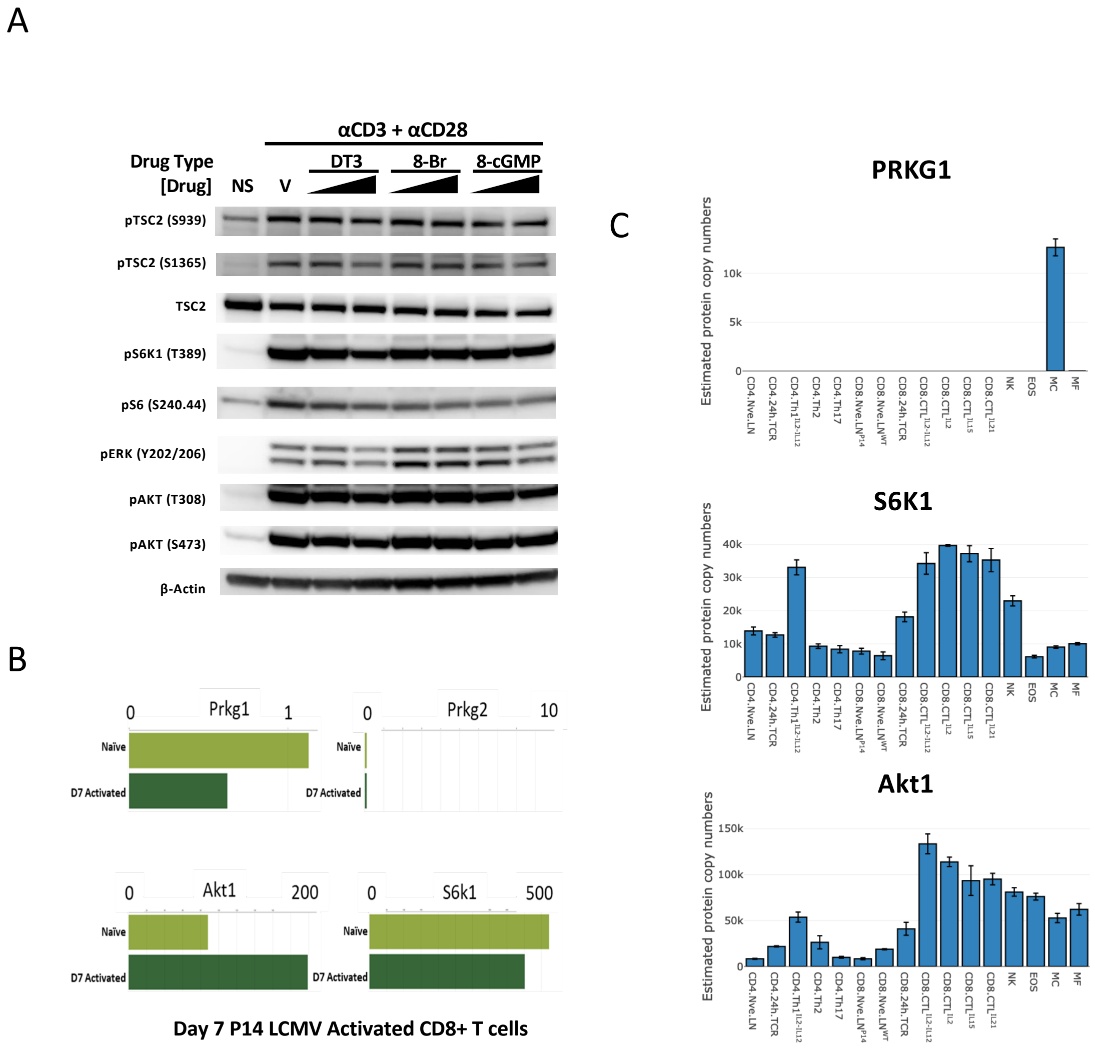


Supplemental Figure 3: **cGMP and cGK-1 do not play a role in T cells. A)** Immunoblot analysis of CD4+ T cells with TCR stimulated by αCD3 with co-stimulation with αCD28, and exposed to increasing concentrations of cGMP agonists: 8-bromo cyclic GMP (8-Br), and 8-pCPT-cGMP (8-cGMP), or with the cGMP-dependent kinase (cGK-1) inhibitor DT3). Neither cGK-1 inhibition or activation impacted TCR+co-stim mediated pTSC2 at S1365. Replicated x2. **B)** Immgen database analysis of Prkg1, Prkg2, Akt1, and S6K1 mRNA expression levels in naïve and D7 activated CD8+ T cells shows virtually no expression of Prkg1 (gene for cGK-1). **C)** Proteomics analysis of PRKG1 (protein known as cGK-1 and PKG1, protein kinase G-1), AKT1, and S6K1. There is no detectable expression of PRKG1 compared to the other kinases.

**Supplemental Figure 4**

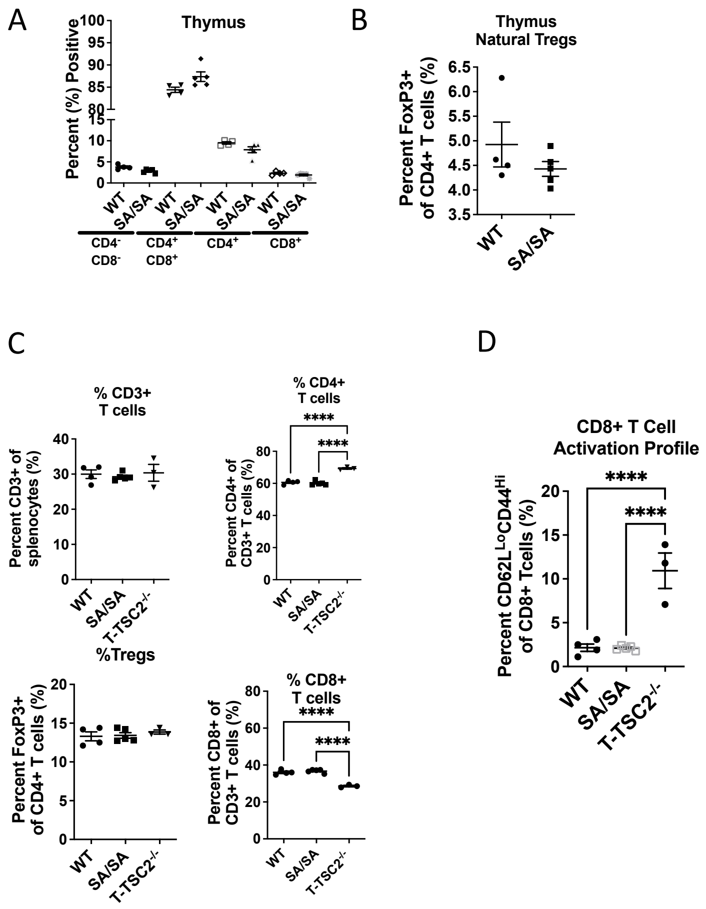


Supplemental Figure 4: **Characterization of lymphocyte development and maturation of age and sex matched WT, TSC2 (SA/SA), and T-TSC2-/-(TSC2 KO) mice. A)** Lymphocyte development and maturation of age and sex matched WT and TSC2 (SA/SA) mice. Flow cytometric phenotyping data compiled from multiple mice (n=4-5) of double negative (CD4-CD8-), double positive (CD4+CD8+), or single positive (CD4+ or CD8+) thymocytes. No significant differences were identified between WT or SA/SA mice. **B)** Flow cytometric phenotyping data of thymic Tregs. Here too, there is no significant difference between the WT and SA/SA genotypes. **C)** Flow cytometric phenotyping data compiled from multiple mice (n=3-5) of percent CD3+, CD4+, Foxp3+ (CD3+CD4+), CD8+ lymphocytes in the spleen. There are no differences between WT and SA/SA. Though a significant rise in %CD4+ and reduction in %CD8+ T cells in TSC2 KO compared to the other two groups. **** P<0.0001. **D)** Flow cytometric phenotyping data of activation profile defined by $CD62L^{Lo}CD44^{Hi}$ on CD3+CD8+T cells in spleen. Unlike T-cells lacking TSC2, this profile remains at WT levels in the SA/SA mice.

# Supplemental Figure 5

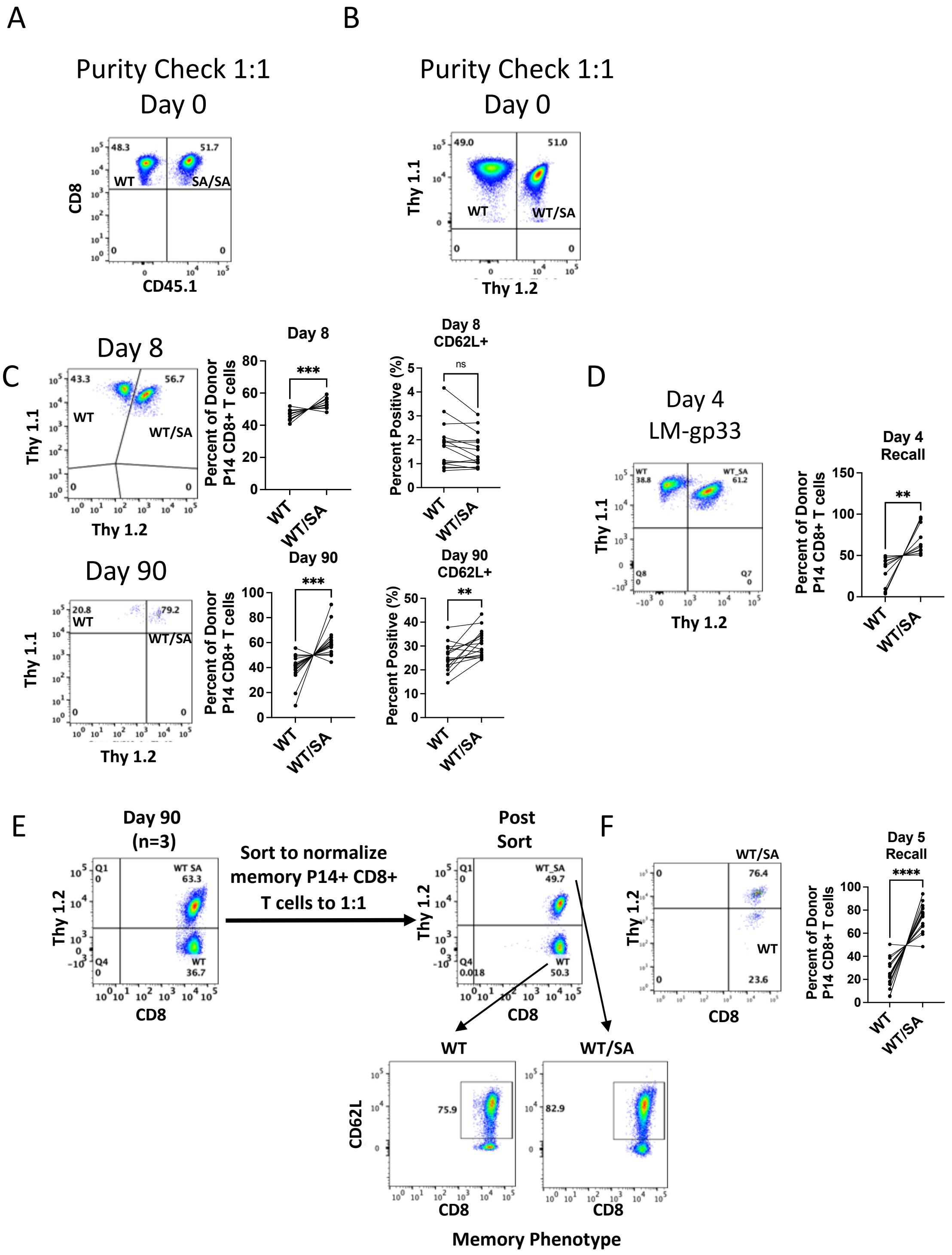

Supplemental Figure 5: **Mutating TSC2 at S1365 (SA) promotes strong effector CD8+ T cell response. A)** Display of pre-transfer check (e.g. equal numbers of CD8+ T cells) for co-adoptive WT and TSC2 SA/SA donor OTI CD8+ T cell study presented in Figure 4A. B-D) WT and mutant TSC2 (SA) transgenic P14 CD8+ T cells co-adoptively transferred into naïve WT hosts followed by acute LCMV Armstrong pathogen infection. **B)** Pre-transfer check of equal representation of co-adoptive WT and TSC2 WT/SA donor P14 CD8+ T cells **C)** Flow cytometry plot of transferred P14 CD8+ T cells (top) and summary data (bottom) showing percent of WT vs WT/SA genotype from donor population 8 and 90 days in blood after exposure to LCMV Armstrong. **D)** Cohort of mice from C were reinfected with Listeria-gp33 for memory recall. Graphical summary of WT and SA mutant P14 CD8+ T cells 4 days after infection in spleen **$p<0.01$ n=10. **E)** Percent of memory WT and mutant TSC2 WT/SA P14 CD8+ T cells in spleen (left, combined n=3). An equal number of memory donor P14 CD8+ T cells were sorted and again co-adoptively transferred into naïve WT recipients to assess memory recall ability on 1:1 basis upon LCMV Armstrong infection. **F)** Percent of donor P14 CD8+T cells after Day 5 upon recall. ****$p<0.0001$. A paired T-test was performed for statistical analysis. Data are representative of at least 2 independent experiments.

# Supplemental Figure 6

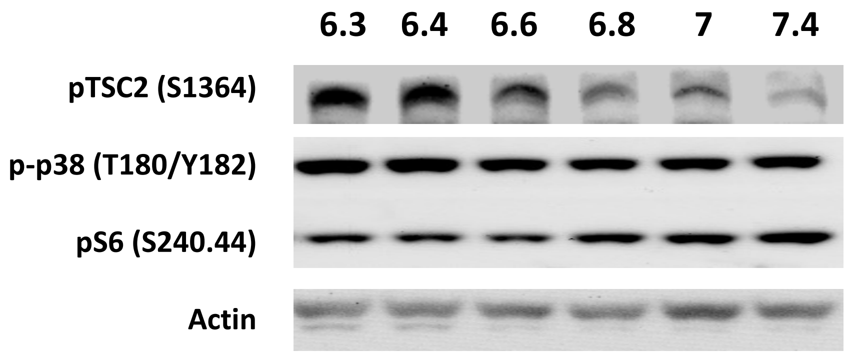


Supplemental Figure 6: **Acidosis results in increased TSC2 S1365 and p38 MAP kinase phosphorylation but reduced mTORC1 activation (pS6) in human T cells.** T cells were incubated at varying pH spanning neutral to moderate acidosis. Immunoblot for pS1365 TSC2, p-p38 and pS6 are shown; actin used for protein loading control.

## Supplemental Figure 7

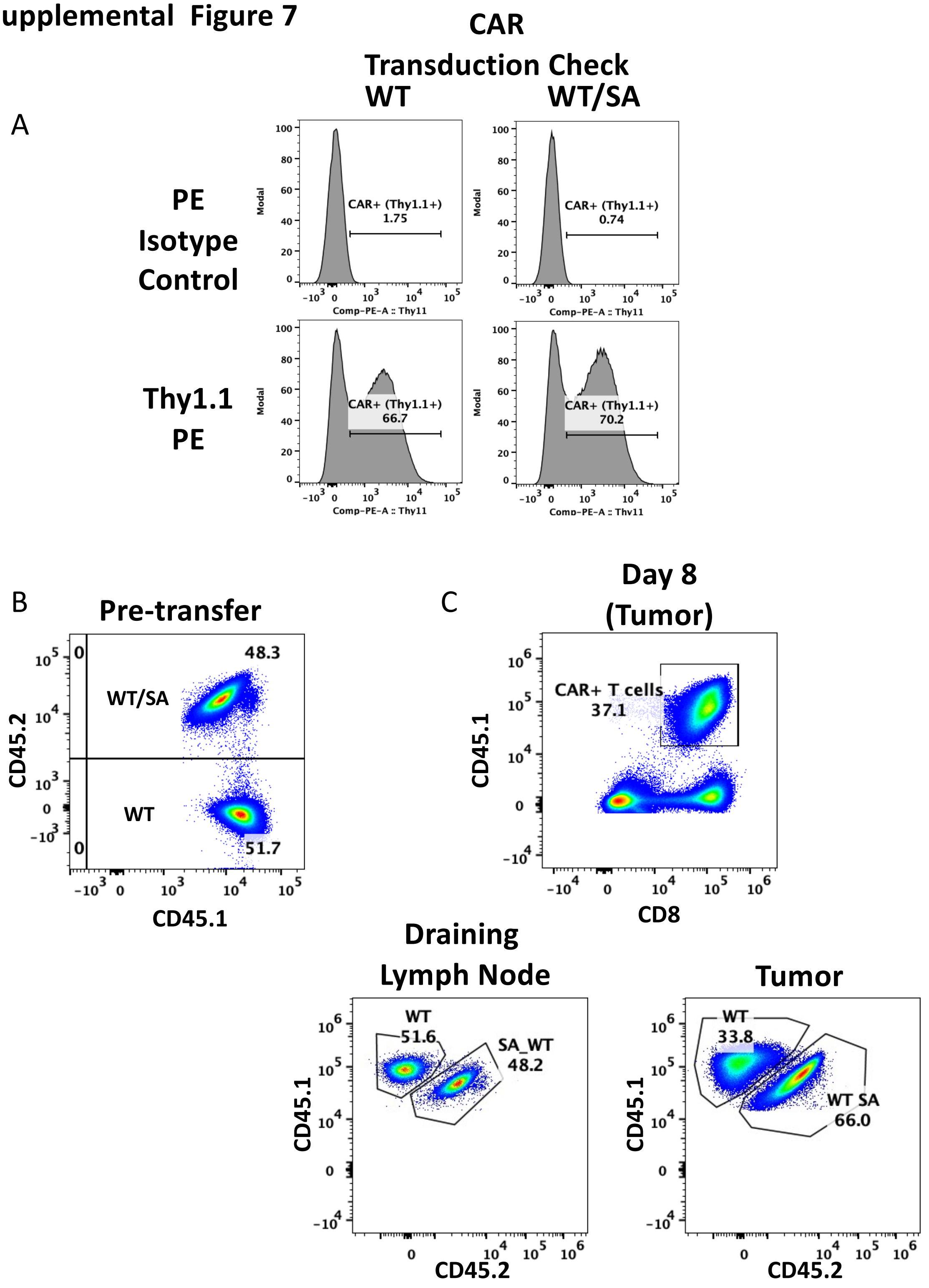

Supplemental Figure 7: **SA mutation improves murine CD19 CAR T cells for adoptive cell therapy. A)** Transduction efficiency of CAR surface expression assessed by Isotype or Thy1.1 expression via flow cytometry in Figure 6 between WT and TSC2 SA mutant CD8+ T cells. **B)** Data show pre-transfer mixture (1:1) of both WT-TSC2 and SA-TSC2 CAR-T cells against human CD19 (hCD19) that were transferred into B16-hCD19 tumor bearing mice. This is then used to compare their presence in tumors, draining lymph nodes, and profile their exhaustion phenotype (Figure 6E). **C)** Example flow-cytometry results summarized in Figure 6E. Donor TILs in B16-CD19 tumors (Day 8) favor SA TSC2 mutation whereas WT and SA are equally represented in draining lymph nodes. Data are representative of at least 2 independent experiments.

## Supplemental Figure 8

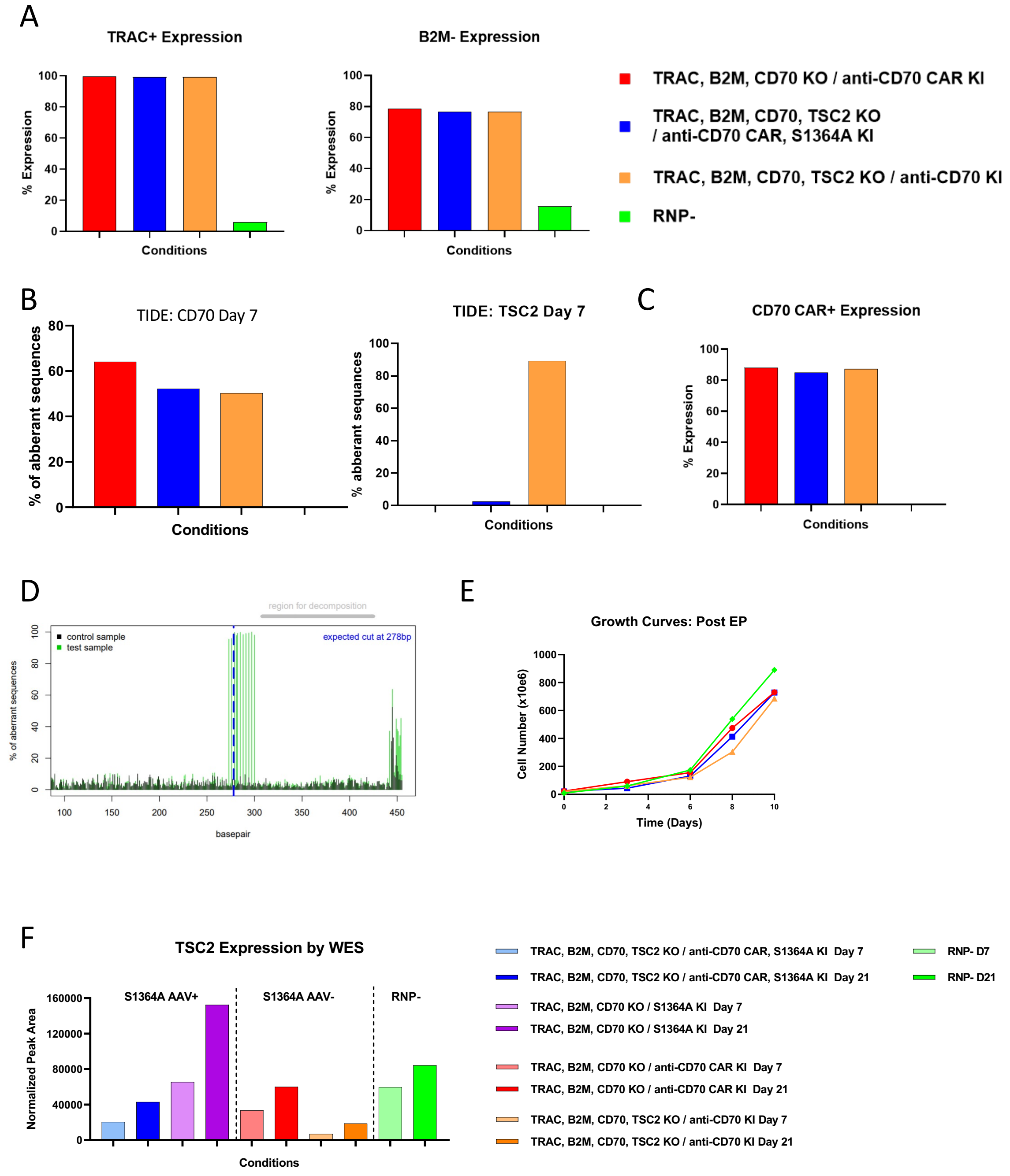

Supplemental Figure 8**: Engineering CD70 CAR T cells with TSC2 (S1364A) mutation. A)** TRAC and B2M knockout was verified by FACS. In cells in which the TRAC loci were edited, the cell populations showed TRAC knock out in less 99.6% of cells, and 79% B2M knock-out surface expression by flow cytometry. **B)** TIDE (tracking of indels by decomposition) analysis verification of CD70 and TSC2 disruption by TIDE analysis. **C)** CD70 CAR expression. **D)** TIDE analysis with S1363A donor template (~100% detection of mutation). **E)** Growth kinetics of CD70 CAR-T cells that were expanded for 10 days. **F)** Western blot analysis to confirm TSC2 deletion. Data was performed one time (A-F)

# Supplemental Figure 9

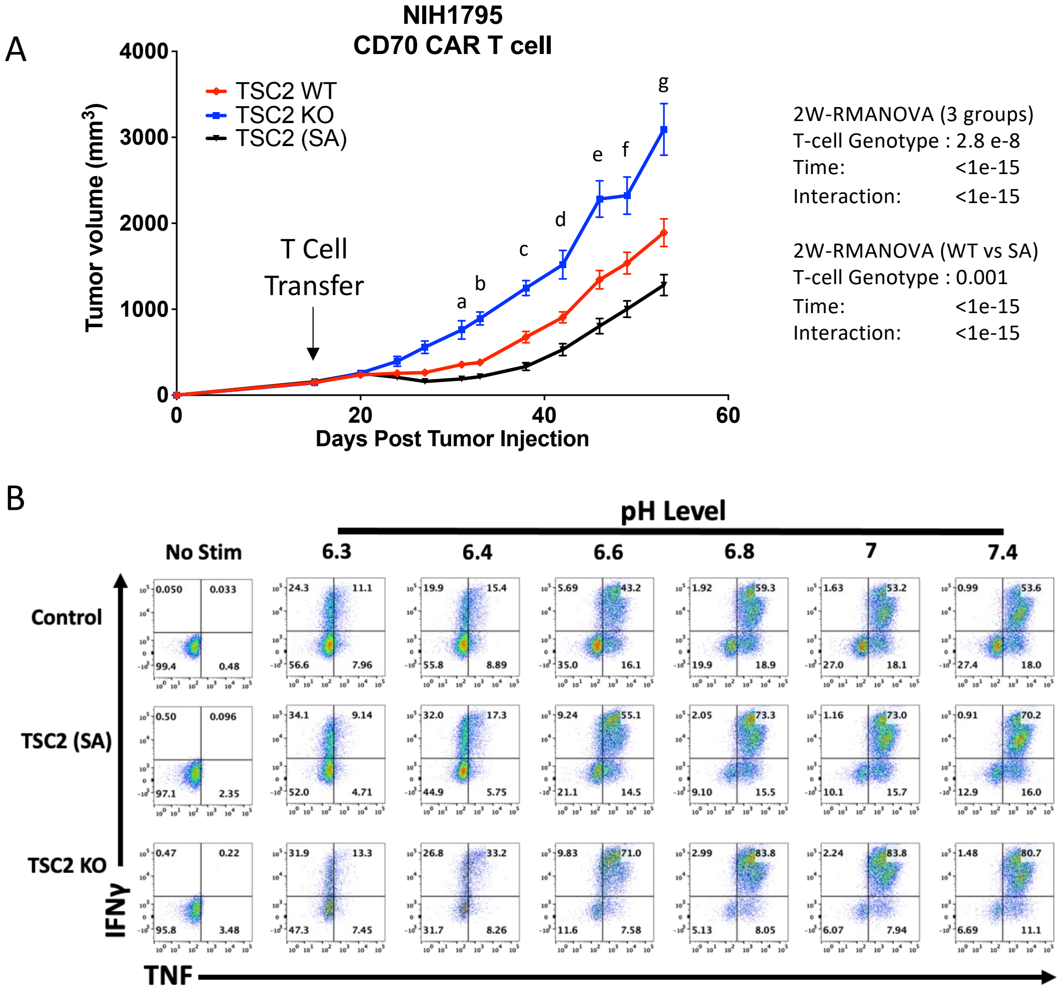

Supplemental Figure 9: **SA mutation improves human CD70 CAR T cell therapy to tumor.**
**A)** Growth curves in NSG mice with that received less CD70 CAR T cells. The dose of cells provided was ~50% below that for the studies shown in Figure 6G. P values for 2way repeated measures ANOVA (2W-RMANOVA) for genotype, time, and interaction are provided. Symbols are Sidaks post hoc multiple comparisons test: P values in table for comparisons between groups at different labeled times.

| Time Comparison | TSC2 KO vs WT | TSC2 KO vs SA | TSC2 SA vs WT |
|---|---|---|---|
| a | | 0.001 | |
| b | 0.006 | 0.00004 | |
| c | 0.001 | 3e-9 | 0.025 |
| d | 0.0003 | 1e-10 | 0.008 |
| e | 5e-10 | <1e-15 | 5e-6 |
| f | 4e-7 | <1e-15 | 6e-6 |
| g | <1e-15 | <1e-15 | 1e-6 |

**B)** Control TSC2 SA/SA, or $TSC^{-/-}$ CD8+ T cells were stimulated with PMA and Ionomycin in media with varying pH levels and IFN-γ and TNFα levels assessed by flow cytometry.
Data was performed one time (A) and two independent times (B)
